# Deconvoluting the Composition of Low-Frequency Hepatitis C Viral Quasispecies: Comparison of Genotypes and NS3 Resistance-Associated Variants between HCV/HIV Coinfected Hemophiliacs and HCV Monoinfected Patients in Japan

**DOI:** 10.1371/journal.pone.0119145

**Published:** 2015-03-06

**Authors:** Masato Ogishi, Hiroshi Yotsuyanagi, Takeya Tsutsumi, Hiroyuki Gatanaga, Hirotaka Ode, Wataru Sugiura, Kyoji Moriya, Shinichi Oka, Satoshi Kimura, Kazuhiko Koike

**Affiliations:** 1 Department of Internal Medicine, Graduate School of Medicine, University of Tokyo, Bunkyo, Tokyo, Japan; 2 AIDS Clinical Center, National Center for Global Health and Medicine, Shinjuku, Tokyo, Japan; 3 Department of Infectious Diseases and Immunology, Clinical Research Center, Nagoya Medical Center, Nagoya, Japan; 4 Director, Tokyo Teishin Hospital, Tokyo, Japan; President, Tokyo Health Care University, Tokyo, Japan; University of Pisa, ITALY

## Abstract

Pre-existing low-frequency resistance-associated variants (RAVs) may jeopardize successful sustained virological responses (SVR) to HCV treatment with direct-acting antivirals (DAAs). However, the potential impact of low-frequency (∼0.1%) mutations, concatenated mutations (haplotypes), and their association with genotypes (Gts) on the treatment outcome has not yet been elucidated, most probably owing to the difficulty in detecting pre-existing minor haplotypes with sufficient length and accuracy. Herein, we characterize a methodological framework based on Illumina MiSeq next-generation sequencing (NGS) coupled with bioinformatics of quasispecies reconstruction (QSR) to realize highly accurate variant calling and genotype-haplotype detection. The core-to-NS3 protease coding sequences in 10 HCV monoinfected patients, 5 of whom had a history of blood transfusion, and 11 HCV/HIV coinfected patients with hemophilia, were studied. Simulation experiments showed that, for minor variants constituting more than 1%, our framework achieved a positive predictive value (PPV) of 100% and sensitivities of 91.7–100% for genotyping and 80.6% for RAV screening. Genotyping analysis indicated the prevalence of dominant Gt1a infection in coinfected patients (6/11 vs 0/10, *p* = 0.01). For clinical samples, minor genotype overlapping infection was prevalent in HCV/HIV coinfected hemophiliacs (10/11) and patients who experienced whole-blood transfusion (4/5) but none in patients without exposure to blood (0/5). As for RAV screening, the Q80K/R and S122K/R variants were particularly prevalent among minor RAVs observed, detected in 12/21 and 6/21 cases, respectively. Q80K was detected only in coinfected patients, whereas Q80R was predominantly detected in monoinfected patients (1/11 vs 7/10, *p* < 0.01). Multivariate interdependence analysis revealed the previously unrecognized prevalence of Gt1b-Q80K, in HCV/HIV coinfected hemophiliacs [Odds ratio = 13.4 (3.48–51.9), *p* < 0.01]. Our study revealed the distinct characteristics of viral quasispecies between the subgroups specified above and the feasibility of NGS and QSR-based genetic deconvolution of pre-existing minor Gts, RAVs, and their interrelationships.

## Introduction

The most recently published study revealed that approximately 180 million individuals are chronically infected with hepatitis C virus (HCV) worldwide [1]. HCV is a positive-sense, single-stranded RNA virus belonging to the *Flaviviridae* family, genus *Hepacivirus*, causing chronic hepatitis C, liver cirrhosis, liver failure and hepatocellular carcinoma [2]. Until recently, the standard therapy for patients infected with HCV have been a combination therapy of pegylated-interferon (peg-IFN) plus ribavirin for up to 48 weeks, in which the sustained virological response (SVR) was achieved in about 50% of the patients even in those infected with hard-to-treat genotypes (including Gt1a and Gt1b). Despite its clinical efficacy, deleterious and nonetheless often common adverse effects, including hemolytic anemia, depression and autoimmune diseases, lead to treatment discontinuation [3]. However, the development of new orally administered therapeutic agents called direct-acting antivirals (DAAs) markedly transformed the treatment against chronic HCV infection into a highly successful one with less severe side effects [4]. Currently available DAAs are roughly classified into three categories on the basis of their molecular target; NS3 protease inhibitors (PIs), NS5A inhibitors and NS5B RNA-dependent RNA polymerase (RdRp) inhibitors [5]. Multiple clinical trials have demonstrated the safety and efficacy of the combined regimens of DAA and peg-IFN and/or ribavirin [6,7]. Moreover, rapidly accumulating evidence suggests the feasibility of a highly effective IFN-free therapy [8–10].

However, the resistance to DAAs might jeopardize the success of HCV treatment. HCV and other RNA viruses exhibit a significant genetic heterogeneity known as quasispecies owing to their extremely error-prone replication [11,12]. This preexisting diversity allows viruses to rapidly develop resistance to antivirals and to escape from host immunity, which may lead to treatment failure. Consistent with their self-diversifying nature, many studies have confirmed the prevalence of naturally occurring resistance-associated variants (RAVs) against DAAs in treatment-naïve HCV patients [13,14]. Indeed, the precise effect of preexisting RAVs on DAA treatment outcome remains elusive, most probably owing to the difficulty in detecting preexisting minor RAVs with sufficiently high accuracy. Nonetheless, the impact of those harbored viral subpopulations should not be undervalued. Even a low-frequency drug-resistant quasispecies at the baseline with an estimated abundance range of 0.07–2.0% has been associated with early therapeutic failure in a study of human immunodeficiency virus (HIV) [15]. As for HCV, one recently published large-scale sequence meta-analysis across five clinical trials showed the association of the NS5B substitutions L159F and V321A with the failure of treatment with sofosbuvir, a potent NS5B RdRp inhibitor [16]. Although the authors concluded that the associated NS5B variants emerged in 2.2–4.4% of patients in whom sofosbuvir treatment failed, the reasons for the treatment failure in the remaining patients were unclear. The limitation of their study is that the detection thresholds of their analyses were set at frequencies of 1–10%. Another study of HCV demonstrated that minor quasispecies at the baseline with the minimum abundances of 0.004–0.02% could in some cases be phylogenetically linked to predominant quasispecies after the failure of peg-IFN plus ribavirin treatment [17]. In a study using chimeric mouse model, harbored quasispecies with RAVs at a frequency of approximately 0.5% became dominant after the failure of DAA therapy, and it has been demonstrated that sequential use of three different classes of DAAs led to the occurrence of triple resistance [18]. Because most resistance-relevant mutations are considered to initially appear as minor variants constituting approximately 0.01–1.0% of the total population, high sensitivity and accuracy are considered prerequisites for analyzing the effect of preexisting minor quasispecies on treatment outcome.

Recent improvements of next-generation sequencing (NGS) enable us to analyze mixed genomic samples in an unprecedented scale. Roche 454 pyrosequencing, which is a pioneer of NGS technology, has been most widely used in viral genomics because of its relatively long read length (∼500 nt), whereas Illumina flow-cell deep sequencing has emerged as a promising alternative owing to its prodigious data productivity and accurate base calling.

Although a hopeful technology, current NGS has several pitfalls. One problem is the difficulty in distinguishing true low-frequency mutations from sequencing artifacts. Because NGS produces a tremendous amount of data, even rare artificial substitutions and indels introduced by the polymerase could distort the interpretation of results, particularly when examining low-frequency single amino acid mutation. A high coverage and a low error rate are highly preferred to circumvent this perplexing problem. Loman et al. compared the sequence accuracy between various benchtop NGS sequencers, concluding that Illumina MiSeq showed the highest throughput per run (1.6 Gb/ run) and lowest error rates (∼0.001 errors per base) [19]. However, since exact error rates are sensitive to experimental condition and target sequences, at present, preliminary simulations and/or control experiments would be indispensable to determine the error rate and detection limit for a true positive variant.

Another problem is that, owing to inestimable viral diversity, conventional reference sequence-based single-nucleotide variant (SNV) detection would be inappropriate for viral research. To overcome this limitation, the bioinformatics approach known as “quasispecies reconstruction (QSR)” is vigorously studied [20–24], in which a vast amount of short and fragmented sequence data is summarized into nucleotide haplotypes (representing viral quasispecies) and their relative abundances, so that even RAVs with a high genetic barrier (amino acid changes derived from two or three concatenating nucleotide substitutions) can be reliably detected. Moreover, this nucleotide haplotype information can be diverted to geno/subtyping, thereby allowing integrated analysis of genotype (Gt) and RAV. Despite a promising strategy for a high-throughput, error-reduced RAV detection, QSR yet poses some challenges to overcome. First, it should be validated using both simulations and clinical samples on a case-by-case basis, as it is yet an emerging and developing technology. Second, the input reads must be sufficiently long and at the same time generated at a high coverage [21]. Schirmer et al. have characterized several types of QSR software with simulated NGS read datasets emulating Roche 454 pyrosequencing (492 nt on average) and Illumina NGS (75 nt), and concluded that QSR did not work properly for Illumina sequence data owing to its relatively short read length [23], although the situation now changes with the recently released Illumina MiSeq reagent v3 allowing a read length of up to 2 x 300 nt. Finally, bioinformatics pipelines must be easily accessible to non-bioinformaticians including physicians and wet-experiment researchers.

In this study, we attempted to characterize and validate QSR-based genotyping and RAV screening pipelines with regards to HCV for analyzing the association between Gts and RAVs. We used preserved serum samples from HCV monoinfected patients, half of whom have a history of blood transfusion, and HCV/HIV coinfected hemophiliacs, who were highly suspected of having multiple exposures to unheated coagulation factor concentrates presumably contaminated with viruses. Illumina MiSeq 2 x 300 nt paired-end deep sequencing was utilized with the reagent v3. QSR was performed using two different types of publicly available, OS-independent software (QuRe and QuasiRecomb) [25,26], and outputs were integrated to achieve better genotyping and RAV screening performance. For genotyping, both the core and NS3 protease region were chosen as targets, whereas only the NS3 protease region was targeted for RAV screening. Preliminary simulation experiments demonstrated that, by combining those two QSR approaches, high sensitivity and positive predictive values could be accomplished at least semiquantitatively with the relative abundance range of around 1.0–99%. Moreover, the genotyping results of clinical samples indicated that, as expected, multi-geno/subtype overlapping infection was common among HCV/HIV coinfected hemophiliacs and HCV monoinfected patients with a history of whole-blood transfusion. Finally, the integrated analysis for Gts and RAVs suggested the possible prevalence of a previously unrecognized linkage including Gt1b-Q80K in NS3 protease regions among HIV coinfected hemophiliacs. This small-scale study illustrated the potential of NGS and QSR-based genotyping and RAV screening, therefore warranting further studies with a larger number of samples to validate the tendencies observed in this study, and to determine the extent to which the response to DAA therapy would be impaired by the preexisting minor variants. Application of our framework to the other HCV genome region such as NS5A and NS5B may also be feasible and helpful for future DAA therapy.

## Materials and Methods

### Patients and Clinical Samples

Ten serum samples randomly selected from HCV monoinfected patients and 11 samples from HCV/HIV coinfected hemophiliacs, all of whom visited our institutions in 2013, were included in this study. All patients were naïve to DAA therapy. All HCV/HIV co-infected patients maintained undetectable HIV RNA by combination anti-retroviral therapy (cART). All HCV/HIV coinfected patients had recurrently used coagulation factor concentrates for hemophilia treatment but no history of blood transfusion. Five HCV monoinfected patients had a history of whole-blood transfusion, whereas the remaining 5 patients did not. The clinical profiles of the patients included in this study are summarized in Table 1. All serum samples were appropriately preserved at −80°C until use.

**Table 1:** Characteristics of this study cohort.

Patient ID	Gender	HIV	BLx ^a^	BLx Background	Clinical Gt^d^
HCVHIV02	Male	+	UCFC ^b^	Hemophilia	1a + 1b
HCVHIV03	Male	+	UCFC	Hemophilia	1b
HCVHIV04	Male	+	UCFC	Hemophilia	1b
HCVHIV05	Male	+	UCFC	Hemophilia	1b
HCVHIV06	Male	+	UCFC	Hemophilia	2a
HCVHIV07	Male	+	UCFC	Hemophilia	1b
HCVHIV10	Male	+	UCFC	Hemophilia	Untyped
HCVHIV11	Male	+	UCFC	Hemophilia	1
HCVHIV15	Male	+	UCFC	Hemophilia	2b
HCVHIV17	Male	+	UCFC	Hemophilia	1b
HCVmono15	Male	−	BT ^c^	Not Available	1
HCVmono17	Male	−	BT	Traffic Accident	1
HCVmono19	Male	−	−	Unknown, BT (−)	1
HCVmono20	Male	−	BT	Burn Injury	1
HCVmono23	Male	−	−	Unknown, BT (−)	1
HCVmono25	Male	−	−	Unknown, BT (−)	1
HCVmono27	Female	−	−	Needlestick Injury	1
HCVmono28	Female	−	BT	Traffic Accident	1
HCVmono29	Female	−	−	Unknown, BT (−)	1
HCVmono34	Female	−	BT	Caesarean section	1

^a^ BLx: Any exposure to blood/blood-related product

^b^ UCFC: Unheated coagulation factor concentrates

^c^ BT: Whole-blood transfusion

^d^ Clinical Gt: Results of clinical genotyping / serotyping

This study was approved by the ethics committees of the University of Tokyo (number 2305-2), and written informed consent was obtained from all study participants in accordance with the Declaration of Helsinki.

### RT-PCR of partial core to NS3 protease region

Viral RNA was extracted from 140 μl of serum using QIAamp Viral RNA Mini Kit (Qiagen, Valencia, CA). RNA was eluted in 60 μl of Buffer AVE, and immediately used for RT-PCR or preserved at −80°C until use.

An aliquot (8 μl) of RNA was reverse transcribed using PrimeScript 1st strand cDNA Synthesis Kit (Takara Bio, Tokyo, Japan). Total RNA was denatured at 65°C for 5 min in a total volume of 10 μl containing dNTPs (1 mM each) and an in-house RT primer (0.2 μM). Denatured RNA was reverse transcribed with 20 units of an RNase inhibitor and 200 units of PrimeScript RTase in a final volume of 20 μl. A reaction mix was prepared on ice, annealed at 30°C for 5 min, reverse transcribed at 42°C for 70 min and stopped at 70°C for 15 min. An aliquot (1 μl) of cDNA was amplified by nested PCR using Expand High Fidelity PCR System (Roche Applied Science, Indianapolis, IN, USA) and in-house primer pairs flanking the 3’ region of the core and the 5’ half of the NS3 protease coding region. The first and second round PCR were carried out in a final volume of 20 μl with 1.5 mM Mg^2+^, 200 μM dNTPs, 0.3 μM forward and reverse primers, 1 U of an enzyme mix and 1 μl of the template. The first round comprised initial denaturation at 94°C for 2 min, followed by 40 cycles of 94°C for 20 sec, 50°C for 30 sec, and 68°C for 4 min. After the 10th cycle, the elongation step was extended in increments of 3 sec per cycle. The final elongation was at 68°C for 20 min. The second round PCR conditions were the same as those of the first round PCR, with the exception of annealing temperature (55°C instead of 50°C). The primers used in this study are listed in S1 Table. An amplicon of around 4.2 kbp was excised from agarose gel and purified using MinElute Gel Extraction Kit (Qiagen), eluted in 20 μl of DNase-free water, and preserved at −20°C for downstream applications.

### Illumina MiSeq next-generation sequencing of partial core to NS3 protease region

PCR amplicons were quantified using a Qubit 2.0 Fluorometer (Life Technologies, Carlsbad, CA) and a 2100 Bioanalyzer (Agilent Technologies, Santa Clara, CA). Paired-end libraries were prepared from 200 ng of DNA using a TruSeq Nano DNA Sample Prep Kit (Illumina, San Diego, CA, USA). Manufacturer’s instructions were strictly followed. Size selection using SPRI beads resulted in DNA ligated with adapters at a size distribution of around 800 bp. Eight cycles of PCR were carried out using barcoded primers, thereby the DNA insert fragment flanked with adapter sequences was enriched. Purified PCR products were pooled so as to contain an equimolar concentration of each library, and 2 x 300 bp paired-end sequencing was carried out using MiSeq and MiSeq Reagent Kits V3 (Illumina).

### Control RNA preparation for estimating MiSeq error rate

Control HCV RNA was prepared by *in vitro* transcription with T3 RNA polymerase (Promega, Madison, WI, USA) and rNTPs (Ambion, Austin, TX, USA) from a linearized plasmid. As a template, a bacterially amplified plasmid (pBSK HC-J1), containing a T3 promoter and a full-length HC-J1 isolate (subtype 1b) sequence (GenBank D10749), kindly gifted by Dr. Tetsuro Suzuki, was used. The prepared RNA was pretreated with TURBO DNase (Invitrogen, Carlsbad, CA, USA), purified using a QIAamp Viral RNA Mini Kit (Qiagen), quantified by NanoDrop spectrophotometer (Thermo Scientific, IL, USA), split into aliquots, and stored at −80°C. Complete digestion of the template plasmid was confirmed by nested RT-PCR omitting the RTase. NGS libraries were prepared in duplicates and sequenced.

### MiSeq data accessibility

Illumina MiSeq sequence datasets (in fastq format) are accessible in the DDBJ Sequence Read Archive (http://trace.ddbj.nig.ac.jp/dra/index_e.html) under the Accession Number of DRA002750.

### Bioinformatics

**Fig 1:**
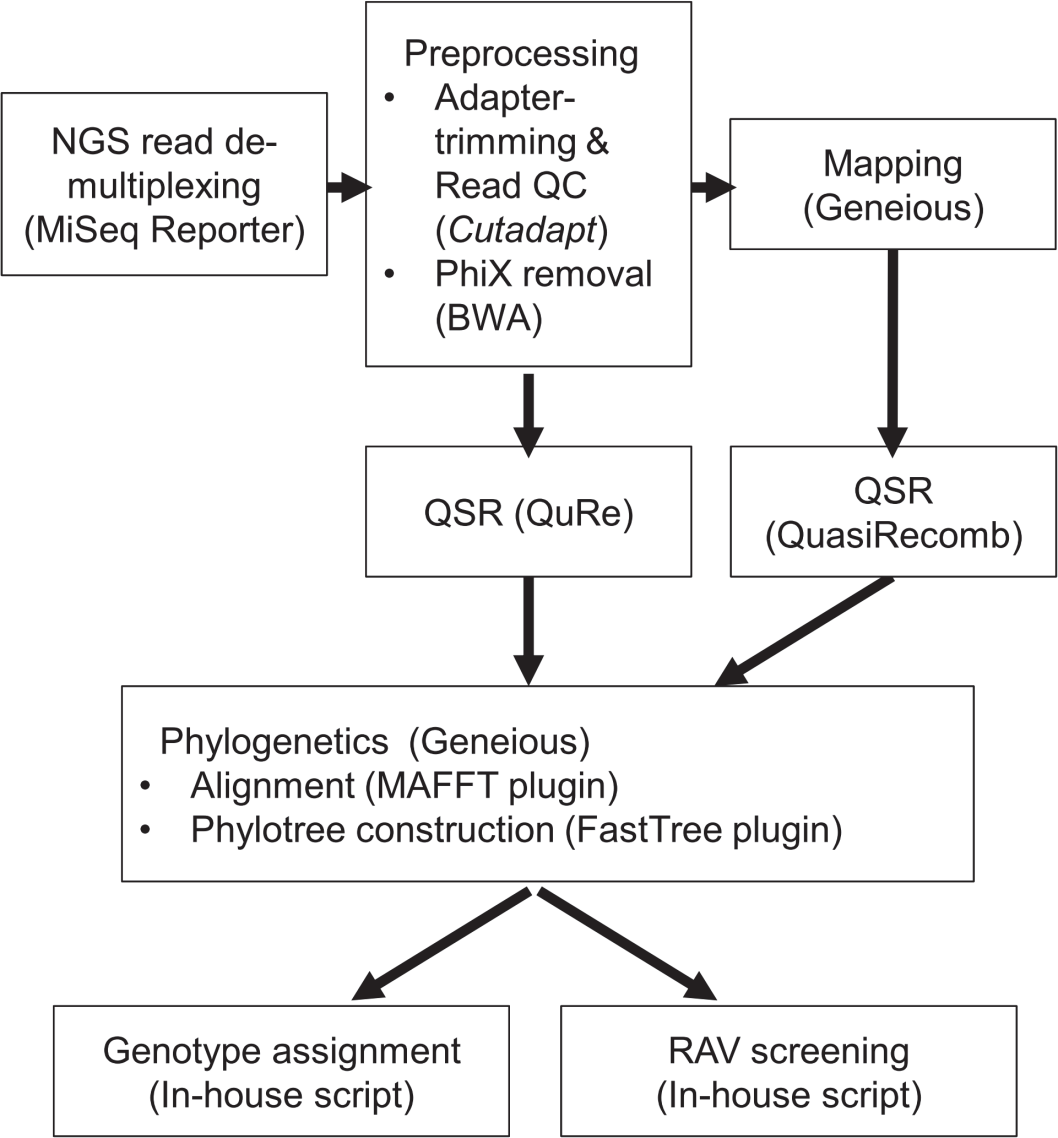
A flowchart of bioinformatics employed in this study.

All sequence analyses were performed using Geneious 7.1 software (Biomatters Ltd., http://www.geneious.com/), sequence analysis suite implemented in Java. All simulations and custom bioinformatics analyses were carried out using Mathematica version 10.0 (Wolfram Research, Inc., http://www.wolfram.com/mathematica/‎) unless otherwise specified. R version 3.1 [27] (http://www.r-project.org/‎) and additional Bioconductor libraries [28] (http://www.bioconductor.org/) were also utilized. All scripts are available upon request. An analysis flowchart was shown in Fig. 1.

### MiSeq read quality control and mapping

Generated reads were adaptor-trimmed using the *cutadapt* program [29] (https://code.google.com/p/cutadapt/). Low-quality reads were removed with a threshold of average quality score < 20. Contaminating PhiX control reads were then removed by mapping to the PhiX reference sequence with the BWA program [30] (http://bio-bwa.sourceforge.net/) with parameters of [-l 20-k 5-n 150]. Unmapped reads were binned using barcode sequences and used for downstream analyses. Mapping to the HCV H77 reference genome (GenBank AF01175) was conducted using Geneious default mapper with the parameter setting of “highest-sensitivity”. Mapping was iterated three times.

### Estimation of pairwise SNV-to-SNV nucleotide distance distribution

Since quasispecies reconstruction (QSR) requires quasispecies-to-quasispecies nucleotide mismatches, or SNVs, as “scaffolds” for concatenating NGS reads that are likely derived from the same quasispecies [20], the distribution of the nucleotide distance between SNVs in any region of interest would be critically important for reliable reconstruction, that is, avoiding the artificial generation of *in silico* recombinants.

Genotype reference sequences were obtained from the HCV Sequence Alignments web tool in The Los Alamos hepatitis C sequence database [31] (http://hcv.lanl.gov/content/sequence/NEWALIGN/align.html) (Alignment type = “Genotype reference”, and Year = “2012”). Obtained sequences were pairwise-aligned using MAFFT [32]. Pairwise SNV-to-SNV nucleotide distance distribution was defined as a set of nucleotide distances between mismatched bases in each pairwise alignment. Pairwise alignments of two reference sequences of the same genotypes were categorized as ‘intragenotype’ and those of the same subtypes as ‘intrasubtype’.

### Quasispecies reconstruction

To simultaneously infer geno/subtype and linked amino acid variants, a conventional SNV calling approach is unsatisfactory. Therefore, an alternative approach, quasispecies reconstruction (QSR), was employed in this study. QSR was performed using both QuRe v0.99971 [25] (http://sourceforge.net/projects/qure/) and QuasiRecomb 1.2 [26] (http://www.cbg.ethz.ch/software/quasirecomb). Since Prosperi et al. [22] previously studied the performance of QuRe, ShoRAH [33] and PredictHaplo (http://cs-wwwarchiv.cs.unibas.ch/personen/roth_volker/HivHaploTyper/index.html), demonstrating QuRe as having a low false positive rate and a reasonably high recall rate among programs compared, ShoRAH and PredictHaplo were omitted from this study. Another set of programs, V-Phaser [34] and V-Phaser 2 [35], were not employed, as these programs can only be used on the Linux platform.

QuRe [25] is a read graph-based, multi-threaded, and platform-independent software implemented in Java. This software requires a long-read (> 100 nt) dataset and a reference sequence for its input. Three calculation steps constitute this software; *k*-mer-based mapping, reconstruction of viral quasispecies sequences and their relative abundances, and a built-in Poisson error correction algorithm, which may also reduce NGS-derived artifacts. In this study, homopolymeric and non-homopolymeric error rate parameters were set to be 0.001, a value taken from a previously reported MiSeq error rate (∼0.001) [19]. A post-reconstruction probabilistic clustering step was omitted. All calculations were iterated 1,000 times. For reference, either the core region or the NS3 protease region of either H77 (GenBank AF01175) or JFH1 (GenBank AB047639) sequence was used. The variant composition in each dataset was reconstructed and output as paired information of sequences and relative abundances.

QuasiRecomb is another QSR software implemented in Java, employing a strategy of probabilistic inference [26]. QuasiRecomb implements a hidden-Markov model for maximum a posteriori (MAP) parameter estimation, automatic model selection and prediction of the quasispecies distribution. It does not require prespecified references, but instead, mapped read set as an input. In this study, BAM files of mapping results generated from Geneious were used. QuasiRecomb also implements many option commands allowing flexible analysis. This time, the flag ‘-conservative’ was not employed because our interest was on minor variants. Either the core or the NS3 protease region was specified using the ‘-r’ command. The variant compositions were reconstructed as with QuRe.

### Genotyping of reconstructed variants

Reconstructed variant sequences were aligned with the Los Alamos genotype reference sequence of either the core or the NS3 protease region using MAFFT [32], and phylogenetic trees were constructed using FastTree [36], both of which tools are implemented as Geneious plugins. Patristic distance matrix was calculated using Geneious from the resultant phylogenetic trees. Each reconstructed sequence was compared with all of the reference sequences, and intra-subtype average patristic distances were calculated using an in-house script. The geno/subtype minimizing the average distance was considered the geno/subtype of the reconstructed sequence.

### RAV screening in NS3 protease region

Simeprevir is a noncovalent, macrocyclic NS3 protease inhibitor [37] and has been proven to be effective in combination with peg-IFN plus ribavirin [38–41] and an IFN-free regimen with sofosbuvir [10]. Despite its efficacy and the mildness of its side effects, there are several RAVs; the amino acid substitutions at V36, F43, Q80, S122, S138, R155, A156, V158, D168 and V170, have been reported to confer resistance against simeprevir [42,43]. Considering its clinical significance, RAVs associated with resistance against simeprevir and relevant DAAs were chosen for screening in this study.

Reconstructed variant sequences were aligned with the NS3 reference sequence using MAFFT, and further codon-aligned and translated using the Codon Alignment v1.1.0 web tool (http://hcv.lanl.gov/content/sequence/CodonAlign/codonalign.html). After gaps were removed manually, relevant amino acid positions were scrutinized using in-house scripts, and the relative abundance of each RAV was calculated.

To assess the performance of QSR-based RAV screening, the SNV-based inference of RAVs was also attempted. BAM-formatted mapping files were used as inputs for the R package deepSNV [44], and SNV frequencies were estimated with the parameters ‘sig.level’ = 0.001 and ‘adjust.method’ = "BH”. As a control counterpart for deepSNV calculation, the MiSeq sequencing data from *in vitro* transcribed control HCV RNA was used.

### Simulation experiments of quasispecies reconstruction

To evaluate the performance of QuRe and QuasiRecomb, *in silico* simulation experiments were carried out. First, MiSeq sequencing files were obtained from three clinical specimens, in which different dominant Gts and amino acid substitutions at NS3 Q80 and/or S122 (Gt1b and Q80K + S122S, Gt1b and Q80Q + S122G, and, Gt2a and Q80G+S122K) were preliminarily identified. Next, mapping was performed, and reads that did not match the dominant substitution were removed. Finally, reads were randomly retrieved from each dataset according to prespecified ratio (see S2 Table) and combined *in silico* into one sequence set. Resultant datasets represent hypothetical quasispecies mixtures of different prespecified relative abundances. In this way, simulation experiments could be performed with sequencing error rates, read length distributions and other characteristics almost the same as the actual NGS. QSR, genotyping and RAV screening were performed as described above. True positives (TPs) indicate the existence of Gts or RAVs specified for simulation, and false negatives (FNs) indicate the failure to detect them. False positives (FPs) indicate the incorrect detection of unintended Gts or RAVs. Sensitivity (Sn) was calculated as the ratio of the number of TPs to the sum of the numbers of TPs and FNs; positive predictive value (PPV) was defined as the ratio of the number of TPs to the sum of the numbers of TPs and FPs.

### Integrated analysis of the association of genotype and RAV for reconstructed quasispecies sequences

Genotyping and RAV screening were carried out for all reconstructed quasispecies sequences as discussed above. Results were then clustered according to (1) the QSR program used, (2) the sample ID, and (3) genotype. If any cluster contained at least one sequence having a specific RAV, the cluster was considered positive for that RAV. In this way, the following attributes were allocated to every cluster: name of QSR software, sample ID, status of HIV coinfection, history of blood exposure (BLx), genotype, and presence or absence of each RAV.

Using this data matrix, univariate and multivariate analyses were conducted to find nominal factors associated with specific RAVs. For univariate analysis, Fisher’s exact test was conducted for each RAV. Significance level was not corrected for multiple testing, and a cut-off threshold was set at an unadjusted *p*-value of < 0.05 for the screening purpose. For multivariate analyses, logistic regression analyses were performed. Significantly associated Gt factors for each RAV were determined by backward stepwise selection with the cut-off threshold of adjusted *p*-value being less than 0.05. In the logistic regression analysis, *p*-values were corrected by Bonferroni’s method, i.e., multiplied by the number of RAVs analyzed.

## Results

### Characterization of Illumina MiSeq NGS reads

The goal of our study was to simultaneously determine the composition of dominant and minor Gts, abundant and low-frequency RAVs, and characteristic combinations of Gts and RAVs from a clinical specimen from an HCV-infected patient. Therefore, we developed an in-house pipeline consisting of (1) NGS data generation, (2) NGS data cleaning, (3) QSR, (4) genotyping and (5) RAV screening of reconstructed sets of sequences, and (6) integration of Gts and RAVs determined from previous analyses of each reconstructed quasispecies.

**Fig 2:**
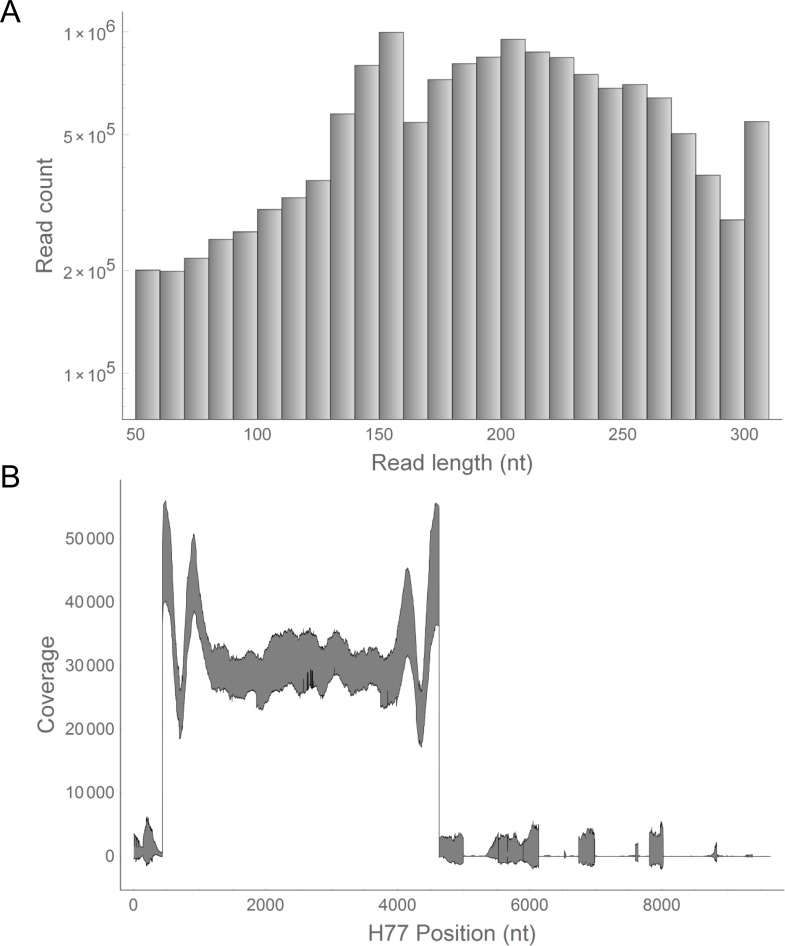
Characterization of Illumina MiSeq sequencing. (A) Read length histogram of all insert sequences of all clinical samples (n = 21). Insert sequences were adaptor-trimmed in advance. (B) Coverage plot showing the 95% confidence intervals of the coverages at all nucleotide positions calculated from all sequence datasets of clinical samples (n = 21). The core region spans from base positions 342 to 914, and the NS3 region spans from 3420 to 5312.

For amplification, RT-PCR was performed using an in-house set of primers (see S1 Table), and the amplicon was excised from agarose gel, purified and analyzed using Illumina MiSeq. From 21 clinical samples, 14,558,762 sequences were obtained after removing low-quality reads and contaminating reads. The length distribution of adaptor-trimmed insert sequences is shown in Fig. 2A; the average length was 194.2 and the standard deviation was 61.0, with the minimum read length of 50 and the maximum of 301. Quality trimming was performed with a threshold of quality score < 20 for each read. The proportion of reads with the least quality score > 30 was 98.0%. Mapping to the HCV H77 sequence was carried out using the Geneious software to confirm uniform coverage (30864 ± 9619 as mean ± s.d.) throughout the amplified region (Fig. 2B).

Next, we estimated the rate of artificial nucleotide substitutions using control RNA (see Methods). The result of SNV screening by deepSNV demonstrated 93 out of 452 nucleotide positions in the HCV core region (463–914 in the genome of the H77 isolate) at the relative abundance range of 0.0145 ± 0.0691 (mean ± s.d.), and only two out of 600 in the upstream region of NS3 protease (3420–4019 in the H77 genome) with their relative abundances of 0.0174 and 0.0298. QSR-based genotyping resulted in Gt1b at an abundance of 1.00. RAV screening revealed no artificial RAVs. S122A was found in one of the duplicates at an abundance of 0.00032, although this variant does not confer resistance.

### Characterization of QSR-based genotyping with simulated datasets

To examine the feasibility of performing QSR on our NGS datasets, we first checked the distributions of nucleotide mismatches between HCV reference sequences. S1 Fig. shows the distributions of SNV-to-SNV nucleotide distances in the core region (base position 463–914 in the genome of the H77 isolate) and the NS3 protease region (3420–4019 in the H77 genome) using HCV reference sequences retrieved from the Los Alamos HCV sequence database. The SNV-to-SNV intervals were significantly shorter than the NGS read length (Mann-Whitney one-tailed tests, *p* < 10^–10^ in all subgroups shown in S1 Fig.).

**Fig 3:**
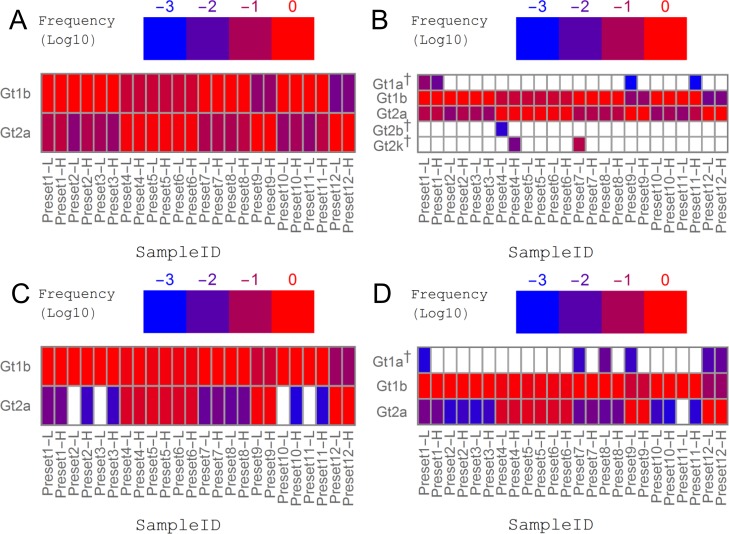
Combination of different QSRs can reduce false-negative genotypes and false-positive genotypes. Simulated datasets were used for QSR calculation followed by genotype (Gt) assignment using either QuRe (JFH1 was used as a reference) or QuasiRecomb. The x-axis labels denote the simulation settings of the preset ratio of relative abundance of intended genotypes (e.g., Gt1b: Gt1b: Gt2a = 95: 95: 5 in the Preset 1 dataset) and the total number of reads (L denoting 30,000 reads, and H denoting 100,000 reads). See S2 Table for all simulation conditions. The y-axis labels are the observed Gts. False-positive Gts (Gts other than Gt1b and Gt2a) are labeled with a dagger (†). (A) Gts observed in both QuRe and QuasiRecomb reconstructions targeting the core region. (B) Gts observed at least once in either QuRe or QuasiRecomb reconstruction targeting the core region. (C) Gts observed in both QuRe and QuasiRecomb reconstructions targeting the NS3 protease region. (D) Gts observed at least once in either QuRe or QuasiRecomb reconstruction targeting the NS3 protease region. From the comparison of the results of QuRe and QuasiRecomb, higher abundances were always selected. The threshold was set at a frequency of 0.001.

Assuming the feasibility of performing QSR on the obtained NGS datasets, we then planned *in silico* simulation experiments with real NGS datasets obtained from clinical specimens. Three samples from HCV/HIV coinfected patients possessing dominant Gt and amino acid substitutions at NS3 Q80 and/or S122 (Gt1b and Q80K + S122S, Gt1b and Q80Q + S122G and Gt2a and Q80G+S122K, respectively) were selected (namely, ‘HCVHIV04’, ‘HCVHIV05’ and ‘HCVHIV06’). NGS read datasets were fabricated by randomly taking reads from three selected sources (see S2 Table for detailed simulation parameters), and QSRs were performed. The genotyping results are summarized in Fig. 3. As for the genotyping of the core region, when only Gts observed commonly in the QSR results of QuRe and QuasiRecomb were retained, expected Gts (Gt1b and Gt2a) were detected under all simulation conditions, whereas no unexpected Gts were retained (Fig. 3A). In NS3, Gt2a (minor Gt) was overlooked in four simulations, in all cases of which the parameter of the total read count was set as low (L). When each Gt observed at least once in the results of either QuRe or QuasiRecomb was retained, unexpected Gts (Gt1a, 2b, and 2k) appeared in the genotyping results of both the core and NS3 (Fig. 3B and 2D, respectively). Erroneously assigned Gts, mainly derived from QuasiRecomb data (data not shown), were equally distant from either Gt1b or Gt2a (S2 Fig.). Seven false-positive Gts were detected in the QSR of the core (Fig. 3B), whereas six false-positive Gts and one false-negative Gt were detected in the QSR of the NS3 protease region (Fig. 3D). Sns and PPVs are summarized in Table 2.

**Table 2:** Properties of QSR-based genotyping.

*Genotyping Method*		TP ^c^	FP ^d^	FN ^e^	Sn ^f^	PPV ^g^
Core	QuRe AND QuasiRecomb ^a^	48	0	0	100.0%	100.0%
	QuRe OR QuasiRecomb ^b^	48	7	0	100.0%	87.3%
NS3	QuRe AND QuasiRecomb ^a^	44	0	4	100.0%	100.0%
	QuRe OR QuasiRecomb ^b^	47	6	1	100.0%	88.7%

^a^ QuRe AND QuasiRecomb: Reproducibly detected by both QuRe and QuasiRecomb

^b^ QuRe OR QuasiRecomb: Detected at least once by either QuRe or QuasiRecomb

^c^ TP: The number of true positives (expected and correctly detected cases)

^d^ FP: The number of false positives (unintended but incorrectly detected cases)

^e^ FN: The number of false negatives (expected but incorrectly overlooked cases)

^f^ Sn: Sensitivity = TP / (TP + FN)

^g^ PPV: Positive predictive value = TP / (TP + FP)

To characterize the quantitative reliability of this genotyping approach, the estimated relative abundance of each Gt was compared with the preset abundance for simulation (S3 Fig.). As for the core region, QuRe reconstructed both dominant and minor Gts quantitatively in all of the simulation conditions tested. Although QuasiRecomb also successfully reconstructed both dominant and minor variants, the abundances of minor variants were more likely estimated to be larger than the preset values, 0.010 and 0.050. QuasiRecomb reconstructed three false-positive Gts at the frequency range of 0.0035 to 0.0497 (S3A Fig.). QuRe also generated three false- positive Gts but their estimated abundances were at the maximum of 0.0013, much smaller than the values of those incorrectly reconstructed by QuasiRecomb (S3B Fig.). In the NS3 protease region, QuRe generated no false-positive Gts but one false-negative Gt under the simulating conditions of a low (L) read count and a preset abundance of 0.010 (S3C Fig.), whereas QuasiRecomb yielded not only one false-negative Gt but also six false-positive Gts at the estimated abundances ranging from 0.0025 to 0.0155 (S3D Fig.).

### Characterization of QSR-based RAV screening using simulated datasets

**Fig 4:**
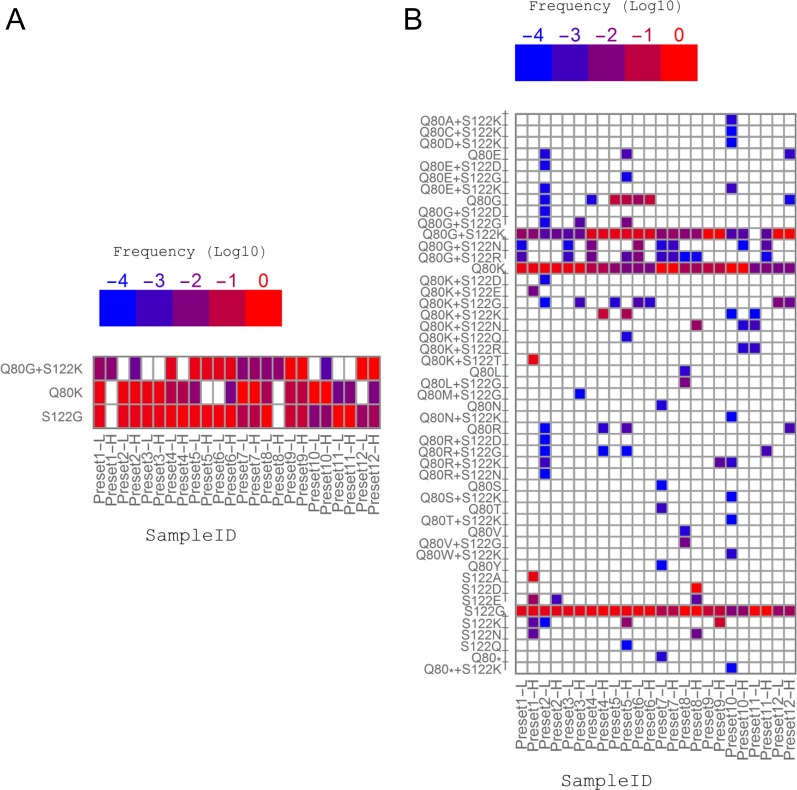
Low-frequency false-positive RAVs can be effectively removed by filtering out variants not reproducibly detected in different QSRs. Simulated datasets were used for QSR calculation followed by the screening of RAVs using either QuRe (JFH1 was used as a reference) or QuasiRecomb. The x-axis labels denote the simulation settings of preset ratio of relative abundance of intended RAVs (e.g., Q80G+S122K: Q80K: S122G = 5: 60: 35 in the Preset 1 dataset) and total number of reads (L denoting 30,000 reads, and H denoting 100,000 reads). See S2 Table for all simulation conditions. The y-axis labels are the observed RAVs. False-positive RAVs (RAVs other than Q80G+S122K, Q80K and S122G) are labeled a dagger (†). (A) RAVs observed in both QuRe and QuasiRecomb reconstructions. (B) RAVs observed at least once in either QuRe or QuasiRecomb reconstruction. From this comparison between the results of QuRe and QuasiRecomb, larger abundances were always selected. The threshold was set at a frequency of 0.0001.

The in-house bioinformatics pipelines for the detection of RAVs in the NS3 protease region were tested using the same simulated datasets discussed above (Fig. 4). When only RAVs reproducibly detected from the results of QuRe and QuasiRecomb were retained, expected RAVs (Q80K, S122G, and Q80G+S122K) were detected with an overall Sn and PPV of 80.6% (58/72) and 100.0% (58/58), respectively (Fig. 4A and Table 3). All of the unexpected RAVs uniquely detected by either QuRe or QuasiRecomb were automatically removed through the consensus-making step. In contrast, when all RAVs observed at least once were kept, Sn increased to 98.6% (71/72) whereas PPV dropped to 41.8% (71/170), apparently owing to the large number of false-positive RAVs (Fig. 4B). We then traced the origin of those false-positive Gts; QuRe had a Sn of 86.1% (62/72) and a PPV of 88.6% (62/70), whereas QuasiRecomb had a slightly higher Sn (93.1%, 67/72) but a much lower PPV (42.9%, 67/156). All the Sns and PPVs are summarized in Table 3.

**Table 3:** Properties of QSR-based RAV screening.

*RAV Screening Method*		TP ^c^	FP ^d^	FN ^e^	Sn ^f^	PPV ^g^
NS3	QuRe AND QuasiRecomb ^a^	58	0	14	80.6%	100.0%
	QuRe	62	8	10	86.1%	88.6%
	QuasiRecomb	67	89	5	93.1%	42.9%
	QuRe OR QuasiRecomb ^b^	71	99	1	98.6%	41.8%

^a^ QuRe AND QuasiRecomb: Reproducibly detected by both QuRe and QuasiRecomb

^b^ QuRe OR QuasiRecomb: Detected at least once by either QuRe or QuasiRecomb

^c^ TP: The number of true positives (expected and correctly detected cases)

^d^ FP: The number of false positives (unintended but incorrectly detected cases)

^e^ FN: The number of false negatives (expected but incorrectly overlooked cases)

^f^ Sn: Sensitivity = TP / (TP + FN)

^g^ PPV: Positive predictive value = TP / (TP + FP)

### QSR-based genotyping with clinical samples

**Fig 5:**
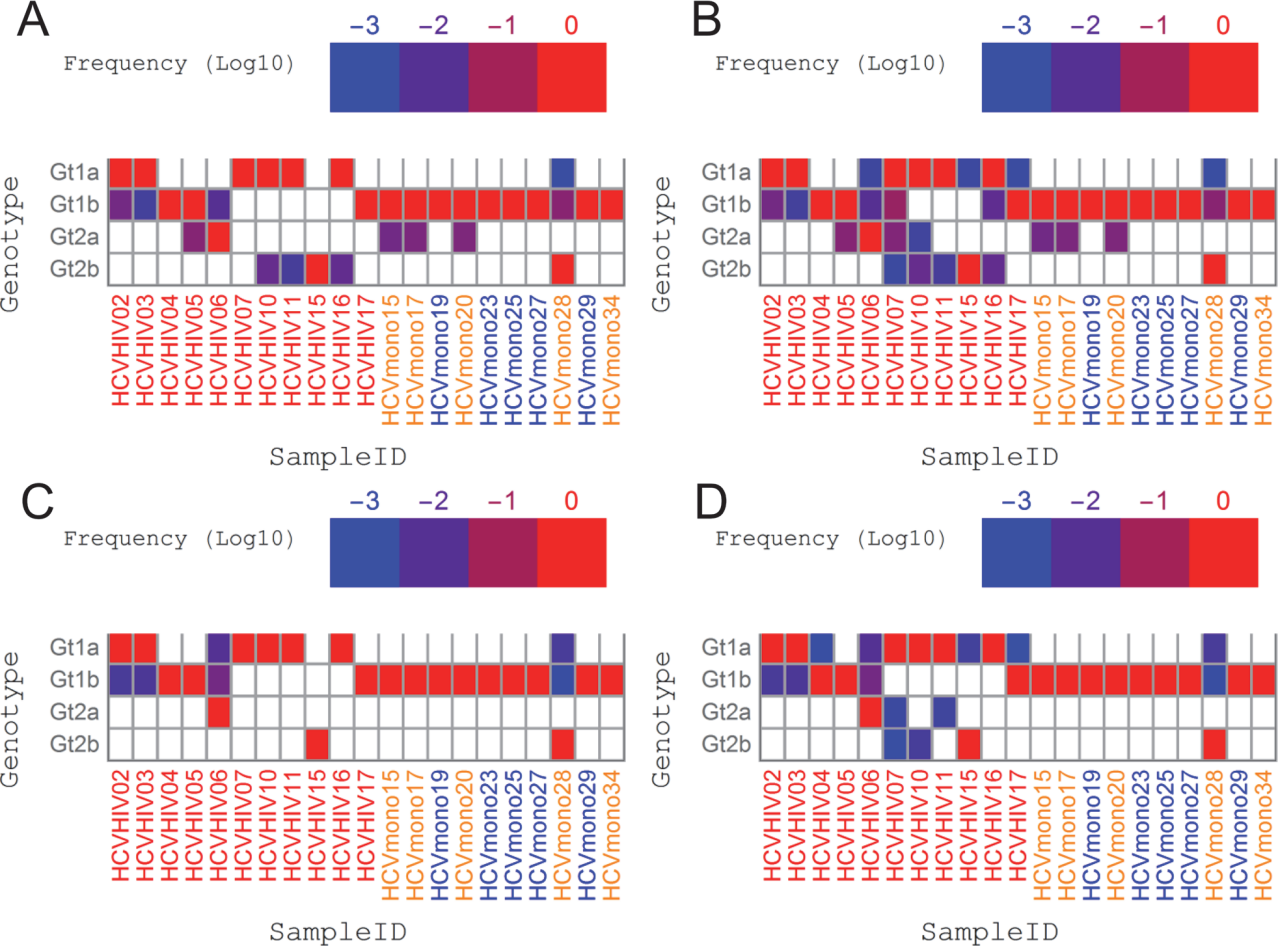
Prevalence of minor multigenotype infections in patients exposed to blood products. Relative abundances of minor genotypes (Gts) were estimated from the genotyping results of reconstructed quasispecies in each subject. The x-axis labels are sample IDs, colored on the basis of the patients’ history of exposure to blood (see Table 1 for details). (A, C) Gts observed in both QuRe and QuasiRecomb reconstructions targeting (A) the core region and (C) the NS3 protease region. (B, D) Gts observed at least once in either QuRe or QuasiRecomb reconstruction targeting (B) the core region and (D) the NS3 protease region. From the comparison of the results of QuRe and QuasiRecomb, larger abundances were always selected. The threshold was set at a frequency of 0.001.

We then attempted to apply our pipelines to the analyses of clinical samples. The genotyping results of 21 HCV-infected patients are summarized in Fig. 5. Because the genotyping strategy of using the core region and taking the consensus of QuRe and QuasiRecomb outperformed other options in the simulation experiments discussed above, we first focused on this strategy (Fig. 5A). Notably, in eight out of 11 HCV/HIV coinfected patients, the dominant Gts were non-Gt1b (6 Gt1a, one Gt2a and one Gt2b), whereas in all but 'HCVmono28' HCV monoinfected patients, Gt1b was dominant. Gt1a infection was dominant only in HCV/HIV coinfected hemophiliacs (6/11 vs 0/10, *p* = 0.0124). Further genotype analysis indicated the presence of multi-geno/subtype overlapping infection in 7 out of 11 HCV/HIV coinfected hemophiliacs and 4 out of 5 HCV monoinfected patients with a history of whole-blood transfusion, whereas none among 5 HCV monoinfected patients without a history of whole-blood transfusion (Fig. 5A). When employing the strategy of incorporating every Gt observed, multi-geno/subtype infection was suspected in 10 out of 11 HCV/HIV coinfected hemophiliacs and 4/5 HCV monoinfected cases with a history of blood transfusion, in an apparent contrast with those cases without a history of blood transfusion (Fig. 5B). The prevalence of multi-geno/subtype overlapping infection was significantly higher in a population with any history of exposure to blood (BLx) (*p* = 0.0124 and *p* = 0.0010 for the genotyping pipeline with or without consensus-based selection, respectively; Fig. 5A and 4B). When the NS3 protease region was used for genotyping, the most dominant genotype estimated in each subject was in good agreement with the genotyping results of the core region. However, overlapping infection was detected in only 3/11 HCV/HIV coinfected patients and 1/10 HCV monoinfected patients when consensus was taken between the results of QuRe and QuasiRecomb (Fig. 5C), and 9/11 HCV/HIV coinfected and 1/10 HCV monoinfected patients when all the genotypes detected by either QuRe or QuasiRecomb-based genotyping were included (Fig. 5D). It was notable that none of the 5 HCV monoinfected patients without BLx had overlapping infection detected by any of the genotyping strategies (Fig. 5).

### QSR-based RAV screening using clinical samples

**Fig 6:**
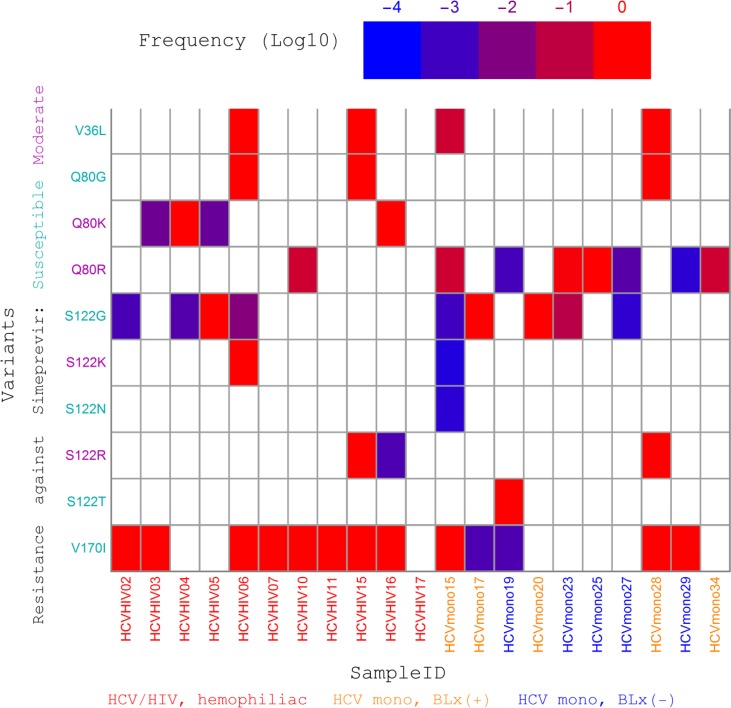
NS3 PI RAVs reproducibly detected by QSR-based screening. Relative abundances of resistance-associated variants (RAVs) in the NS3 protease region were estimated in each subject. Only RAVs reproducibly detected in the QSR results of QuRe and QuasiRecomb were retained. The x-axis labels are sample IDs, colored on the basis of their history of exposure to blood (see Table 1 for details). The y-axis RAV labels are colored on the basis of the effects of RAVs on simeprevir susceptibility: susceptible (FC < 2) substitutions are in cyan, moderately resistant substitutions (2 < FC < 50) in magenta. Highly resistant substitutions (FC > 50) were not detected. The threshold was set at a frequency of 0.0001.

Considering the clinical significance of simeprevir, RAVs associated with resistance against simeprevir and relevant DAAs were chosen for subsequent analyses. Screening results are summarized in Fig. 6. Ten RAVs remained after removing disagreement between the QSR results of QuRe with the H77 sequence as the reference, QuRe with the JFH1 sequence as the reference, and QuasiRecomb. Eight of 10 variants were related to either Q80 or S122. No variants at positions R155, A156, V158, and D168 were definitively proven. It was notable that only 13 variants were detected using QuRe (S4 Fig.), whereas the total number markedly increased to 65 in the case of using QuasiRecomb (S5 Fig.). Q80K was detected in 4 out of 11 HCV/HIV coinfected hemophiliacs, whereas Q80R was detected in 1 out of 11 patients coinfected with HIV and HCV, and 7 out of 10 HCV monoinfected patients (*p* = 0.0075). A set of V36, Q80G, and either S122K or S122R was observed in patients ‘HCVHIV06’, ‘HCVHIV15’, and ‘HCVmono28’, all of whom had Gt2 as the dominant genotype. Low-frequency S122K and S122R were detected in one (‘HCVmono15’) and two (‘HCVHIV16’ and ‘HCVmono28’) cases, respectively.

**Fig 7:**
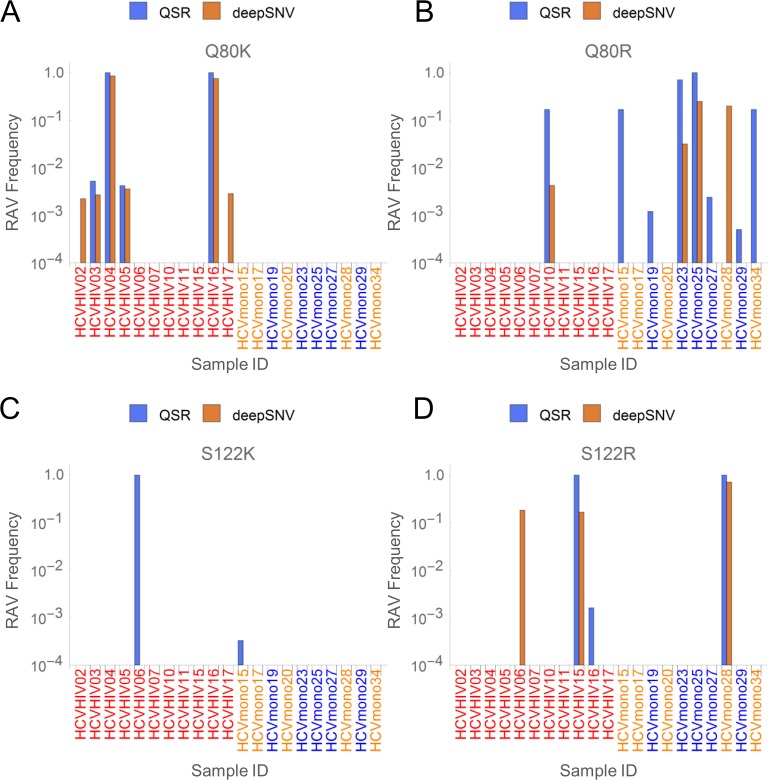
QSR-based RAV screening can detect amino acid changes derived from multiple nucleotide substitutions. The relative abundances of (A) Q80K, (B) Q80R, (C) S122K, and (D) S122R determined by QSR-based RAV screening and inference from SNVs detected by deepSNV (see Method).

Q80 and S122 have been associated with decreased viral sensitivity and treatment failure [42]. Therefore, we decided to focus on Q80K, Q80R, S122K, and S122R, all of which cause resistance against simeprevir with the fold change of more than 2 (considered moderate resistance) in Gt1a and Gt1b. After validating their existence by manually inspecting mapping data (data not shown), we compared estimated abundances of each RAV by (1) QSR-based screening with consensus selection, and (2) SNV-based inference of RAVs (deepSNV), where the R package ‘deepSNV’ was used to estimate the frequencies of relevant SNVs. The results are shown in Fig. 7. Q80K was detected in 4 out of 11 HCV/HIV coinfected hemophiliacs but not in any of the 10 HCV monoinfected patients (Fig. 7A). Q80K was also detected by deepSNV in those 4 cases. However, there were 2 patients in whom Q80K was indirectly inferred on the basis of SNVs but not by the QSR-based screening (Fig. 7A). Q80R was detected in 1 out of 11 HCV/HIV co-infected and 7 out of 10 HCV monoinfected patients. The program deepSNV failed to detect Q80R in 5 out of 8 cases and incorrectly inferred its existence in one case, HCVmono28, wherein the reference codon was CAA, the corresponding variant codon was GGG, and the incorrectly inferred codon was CGA (the responsible SNV is underlined hereafter; Fig. 7B). As for S122 variants, S122K was detected in ‘HCVHIV06’ by QSR (Fig. 7C). The deepSNV-based screening failed to detect S122K (the corresponding codon was AAG) in ‘HCVHIV06’ and incorrectly interpreted the relevant SNVs as S122R (the reference codon was AGC and the incorrectly inferred codon was AGG) (Fig. 7C and 6D). In ‘HCVHIV16’, S122R was detected by QSR but not by deepSNV-based screening. Mapping files were manually checked to confirm the correctness of RAVs detected by QSR (data not shown).

### Integrated analysis of genotypes and RAVs

Because multi-geno/subtype overlapping infection was prevalent in this study cohort, there could be multiple cases wherein observed RAVs should be allocated to quasispecies of different genotypes. Therefore, we aimed at linking the Gts and RAVs. All reconstructed quasispecies sequences were clustered according to the sample ID and assigned genotype, and within each cluster, the presence or absence of RAVs was determined (see Methods). Univariate and multivariate interdependence analyses for 94 clusters revealed that Q80K and Q80R were significantly associated with hemophilia and BLx (Tables 4 and 5). When focusing on HCV/HIV coinfected hemophiliacs, the association between Q80K and Gt1b was statistically significant [odds ratio (OR) = 13.4 (3.48–51.9), *p* < 0.01]. In contrast, when focusing on quasispecies reconstructed from HCV monoinfected patients, Q80K was inclined to have a certain association with Gt1a, although the *p*-value was not below the threshold of statistical significance after correction for multiple testing [OR = 70 (3.10–1580), uncorrected *p* < 0.05]. Univariate analysis indicated a negative correlation between hemophilia and the presence of Q80R [OR = 0.13, *p* < 0.001], although this was not proven by multivariate analysis. On the other hand, S122 variants were not statistically linked with either hemophilia or BLx. However, when the linkage of RAVs on the same reconstructed sequences was taken into consideration, the linked variants of S122K + Q80G were associated with Gt2a [OR = 174 (12.9–2350), *p* < 0.05], and those of S122R + V36L + Q80G were associated with Gt2b [OR = 145.0 (17.7–1190), *p* < 0.001].

**Table 4:** Univariate analysis of nominal factors associated with NS3 Q80K/R and S122K/R.

*Univariate*						
RAV ^a^	Odds ratio					
	Hemophilia ^c^	BLx ^d^	GT1a ^b^	GT1b	GT2a	GT2b
Q80K	5.84 ^††^	Inf ^e^ ^,^ ^†^				
Q80R	0.13 ^†††^	0	0.21 ^††^	3.49 ^††^		
S122K					42.0 ^†††^	
S122R		Inf ^†^		0.23 ^††^		37.3 ^†††^

^a^ RAV: Resistance-associated variant

^b^ Gt: genotype

^c^ Hemophilia: Hemophilias with HCV/HIV coinfection and multiple exposures to unheated coagulation factor concentrates

^d^ BLx: Exposure to unheated coagulation factor concentrates, or history of whole-blood transfusion

^e^ Inf: Infinity

^†^ Uncorrected *p* < 0.05

^††^ uncorrected *p* < 0.01

^†††^ uncorrected *p* < 0.001

**Table 5:** Multivariate analysis of nominal factors associated with NS3 Q80K/R and S122K/R.

*Multivariate*						
RAV ^a^	Odds ratio [95% confidence interval]					
	Hemophilia ^c^	BLx ^d^	GT1a ^b^	GT1b	GT2a	GT2b
Q80K	13.6 [3.14–58.5] *		70 [3.10–1580] ^†^ ^,^ ^##^	13.4 [3.48–51.9] ** ^,^ ^#^		
Q80R	0.13 [0.05–0.32] ***					
Q80R +V36L		0.02 [0.002–0.15] *				
S122K						
S122K +Q80G					174. [12.9–2350] *	
S122R			0.10 [0.02–0.54]^†^ ^,^ ^##^	0.02 [0.002–0.27] ^†^ ^,^ ^##^		44.7 [5.11–390] *
S122R +V36L						624 [35.5–11000] *
S122R +Q80G						273 [21.9–3400] *
S122R +V36L +Q80G						145 [17.7–1190] ***

^a^ RAV: Resistance-associated variant

^b^ Gt: genotype

^c^ Hemophilia: Hemophiliacs with HCV/HIV coinfection and multiple exposures to unheated coagulation factor concentrates

^d^ BLx: Exposure to unheated coagulation factor concentrates, or experience of whole-blood transfusion

^†^ Uncorrected *p* < .05

* *p* < .05

** *p* < .01

***p < .001

^#^ Logistic analysis with only 'Hemophilia' (+) clusters included

^##^ Logistic analysis with only 'Hemophilia' (−) clusters included

**Fig 8:**
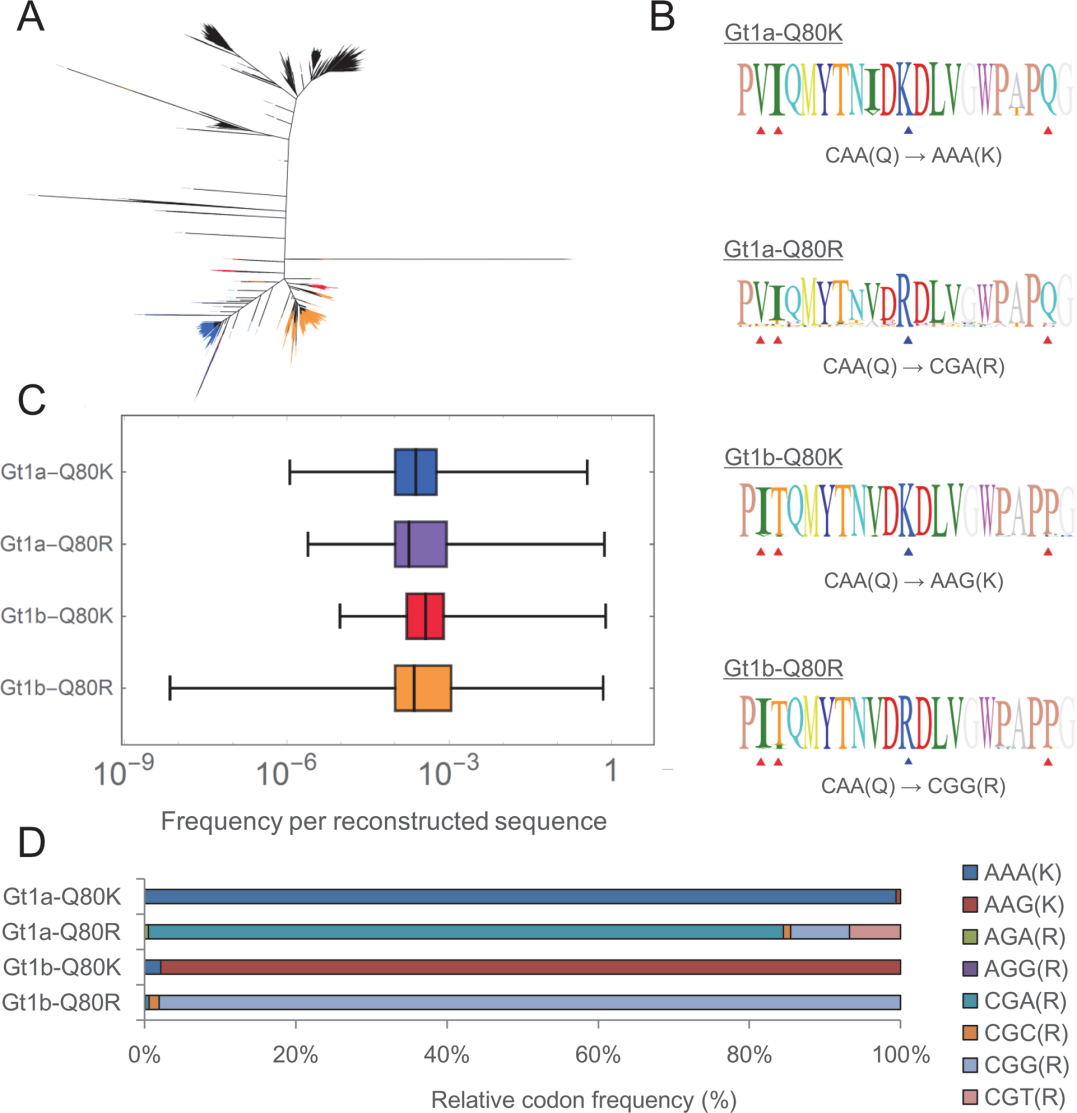
Integrated interdependence analysis of Gt and RAV enables high-throughput identification of distinct subpopulations with characteristic combinations of Gt and amino acid haplotype. (A) Phylogenetic analysis of all reconstructed sequences of NS3 protease region. Taxa are colored on the basis of their assigned Gt and Q80 RAV as follows: Gt1a-Q80K = blue; Gt1a-Q80R = purple; Gt1b-Q80K = red; Gt1b-Q80R = orange. A phylogenetic tree was generated using FigTree software. (B) Sequence logos from all sequences assigned to each pair of Gt and Q80 amino acid variant depicted in (A). Blue triangles denote NS3 Q80. Red triangles denote geno/subtype-specific amino acid polymorphisms at positions 71, 72, and 89. The codon change from reference to the most dominant variant at position 80 was denoted. (C) Distributions of estimated frequency per reconstructed sequence. (D) Relative codon frequencies for each Gt-RAV. The frequency was defined as the ratio of the number of reconstructed sequences possessing each codon and the total number of reconstructed sequences.

We then attempted to further characterize the amino acid linkage, phylogenetics, and the distributions of estimated frequencies of quasispecies sequences having those variants. Q80K and Q80R were focused on in subsequent analyses as these variants were presumably linked with Gt1 infection, a principal target of many DAA therapies such as simeprevir. Quasispecies of Gt1a with Q80K, Gt1a with Q80R, Gt1b with Q80K, and Gt1b and Q80R were detected from combined QSR results of all samples (Fig. 8). A phylogenetic tree was constructed, which indicated a distinct subpopulation with each combination of genotype and Q80 substitution (Fig. 8A). Sequences assigned to each cluster were retrieved, aligned, and visualized as a sequence logo (Fig. 8B). Analyses of amino acids at positions 70–90 revealed that the sequences of Gt1b had Gt1b-specific amino acids, whereas the sequences of Gt1a had Gt1a-specific amino acids at positions 71,72, and 89 (V, I, and Q for Gt1a, and I, T, and P for Gt1b, respectively), regardless of the amino acid variant at position 80. However, Gt1a-Q80R sequences had V78, which is the characteristic of Gt1b. The codon usage patterns at position 80 also differed from one another (Fig. 8B and 8D). The most dominant codons were AAA(K), CGA(R), AAG(K) and CGG(R) and their relative frequencies were 99.4%, 84.0%, 97.9% and 98.1% for Gt1a-Q80K, Gt1a-Q80R, Gt1b-Q80K and Gt1b-Q80R, respectively. Fig. 8C shows the distributions of relative frequency per reconstructed quasispecies sequence having these Gt–RAV pairs. In every Gt–RAV pair, the relative frequencies ranged from ∼0.01% to ∼100% with no remarkable differences.

Furthermore, the Los Alamos HCV sequence database [31] was scrutinized to find previously registered cases of the combinations of Gt1a-Q80K, Gt1a-Q80R, Gt1b-Q80K, and Gt1b-Q80R. All the sequences containing the NS3 region were analyzed, and the registered sequences were binned by genotype and sampling country (S3 and S4 Tables, respectively). Surprisingly, there was only one sequence of Gt1b with Q80K, and there were only three cases of Gt1a with Q80R (S2 Table). Most reports of sequences with Q80K or Q80R were from the United States, and none were previously registered in Japan (S3 Table).

## Discussion

In this study, we developed and characterized the Illumina MiSeq NGS sequencing system coupled with a novel, accurate, and high-throughput bioinformatics pipeline involving quasispecies reconstruction (QSR), genotype (Gt) assignment, screening of resistance-associated amino acid variants (RAVs), and integrative analysis of the association between Gts and RAVs.

Our approach has several novelties compared with those used in previous studies. First, many previous studies have used Roche pyrosequencing-based NGS, not Illumina's flow-cell-based sequencer. Loman et al. characterized the performance of several bench-top NGS sequences, concluding that Illumina MiSeq has the highest read generation capability with the lowest frequency of sequence errors, particularly indels [19]. Because indels and other sequencing errors could result in false-positive low-frequency RAVs, a high read coverage and a low error rate would be preferred in viral research. Next, we employed the QSR technique to (1) eliminate sequencing errors through the reconstruction step, and (2) obtain sets of haplotypes spanning the genome region of interest (mimicking cloning experiments). Although Illumina NGS has not been considered suitable for QSR owing to its short read length, we for the first time successfully applied the QSR technique to NGS reads from MiSeq 2 x 300 nt paired-end sequencing. It was preliminarily confirmed that the SNV-to-SNV intervals were sufficiently short compared with the distribution of NGS reads obtained (Fig. 2B and S1 Fig.). Quasispecies sequences were successfully reconstructed in this study with a sufficient length covering the core region and NS3 protease region. Furthermore, we achieved highly accurate estimations of Gts and RAVs by combining two QSR programs, QuRe and QuasiRecomb, both of which were based on different algorithm principles. Initially, we had a concern about artificial recombination attributed to PCR amplification step and/or QSR calculation step. However, the simulation experiments demonstrated the accuracy of our QSR-based genotyping (Fig. 3 and Table 2) and RAV screening (Fig. 4 and Table 3) without *in silico* RAV recombination proven (Fig. 4A). A high PPV, rather than a high Sn, would be preferable for future investigation, because a high PPV would allow effective selection of patients having “true-positive” low-frequency RAVs, without the annoying false-positive RAVs. This is particularly important for research focusing on the impact of pre-existing minor RAVs, because a considerable number of false-positive RAVs at the preliminary screening stage might lead to a false conclusion that minor RAVs showed no correlation with the treatment outcome. In addition, note that sensitivity is in principle restricted by the coverage depth attainable with the sequencers currently available; therefore, so methodological improvement would be difficult. Lastly, by desterilizing the reconstructed haplotype information, we combined genotyping and RAV screening so as to find a novel relationship between them. The limitations of conventional SNV-based mutation screening are summarized into the following points: (1) it was difficult to gain genotype information; (2) it was difficult to link detected SNVs to correctly infer relevant RAVs, especially when multi-geno/subtype clones co-existed; and (3) it was impossible to gain insight on the basis of epistatic interactions between mutations, which has recently been predicted in HIV protease by a systems approach [45]. Our approach can overcome these limitations, which can reveal how the impact of one mutation depends on the presence or absence of other mutations in the context of clinical trials and post-trial surveys.

Recently, Jabara et al. have reported a novel solution to eliminating errors introduced during PCR amplification and pyrosequencing by using a single-molecular identifier [46,47]. The principle of this strategy is the use of a RT primer tagged by an 8 degenerate ID sequence. The resultant pyrosequencing reads having the same ID tag sequence are clustered, and the consensus sequence is generated on the majority basis, thus enabling the effective removal of artificial errors introduced during PCR, library preparation, and NGS. Polymerase error rate has been vigorously studied because of its potential impact on the inference of viral quasispecies diversity [48]. Although a promising technique, however, the analysis of this diversity could not yet be considered error-free owing to the error-prone nature of reverse transcriptase. The error rate of the commonly used, MMLV RTase was reported to be around 10^−5^–10^−4^ per nucleotide [49], which might still be sufficient to artificially generate low-frequency false positive variants. Moreover, the read length of the NGS sequencer, a maximum of ∼1000 bp achieved using the Roche GS FLX+ system, would be an inevitable limitation of this methodology. Another solution that has recently been described by Acevedo et al. is circular sequencing (CirSeq), wherein circularized genomic RNA fragments are used to generate tandem repeats [50,51]. These repeated reverse transcriptions principally eliminate even the errors introduced by the reverse transcriptase use. The CirSeq approach in principle would provide completely error-free sequencing, but the target RNA must be fragmented into small pieces before amplification, which would be unfavorable for linkage analysis. In contrast to these emerging techniques, our analysis pipeline is much more practical. Moreover, our framework can be applicable even to previous NGS data obtained from ordinary RT-PCR experiments, as long as the read lengths are sufficiently large. NGS sequence meta-analysis is an emerging but promising strategy to integrate our knowledge leading to deeper insights on viral quasispecies dynamics.

Among hemophiliacs frequently receiving coagulation factor concentrates, the prevalence of HCV infection was high (60–90%) [52,53]. Before 1984, preheating was not yet routinely performed during the preparation of coagulation factor concentrates to inactivate contaminating HIV [54,55]. Moreover, blood products were frequently imported from countries overseas including the United States, as there were insufficient blood donors in Japan. Thus, patients using blood products at that time were exposed to the risk of infection with not only HCV but also HIV, which had not yet been prevalent in Japan. Considering this specific circumstance, we hypothesized that there were HCV quasispecies of different genetic and geographic origins among HCV monoinfected non-hemophiliacs and HCV/HIV coinfected hemophiliacs in Japan. As expected, our analyses demonstrated that the compositions of genotypes and RAVs were quite different between HCV/HIV coinfected hemophiliacs, HCV monoinfected patients with previous exposure to whole-blood transfusion (BLx), and HCV monoinfected patients without a history of exposure to BLx. Gt1b was dominant (10 out of 11 = 91%) among cases without HIV coinfection, whereas Gt1a was dominant (6 out of 11 = 54%) among HCV/HIV coinfected patients. The predominant infection with Gt2a and Gt2b was determined in 3 cases. No other genotypes such as Gt3 and Gt4 were detected in this study. Moreover, multi-geno/subtype overlapping infection was significantly more prevalent among hemophiliacs and patients with BLx. This high prevalence of overlapping infection might explain the changes in genotype frequently observed among hemophiliacs [56,57] and other at-risk populations [58]. Furthermore, investigation on the interrelationships between Gts and RAVs suggests that Q80K was more prevalent in HCV/HIV coinfected hemophiliacs, whereas Q80R was less prevalent in HCV monoinfected non-hemophiliacs (Tables 4 and 5). A notable finding is that Q80K was significantly associated with Gt1b quasispecies among the hemophiliacs in this study (Tables 4 and 5, and Fig. 8). The Q80K variant is observed in 5.7–38% and 0.0–0.8% of patients with Gt1a and Gt1b HCV infections, respectively [42]. Q80K confers a 9.3-fold resistance against simeprevir in the Gt1a replicon system [42], and one clinical Phase 2 trial of simeprevir showed reduced SVR 24 rates with patient with Q80K mutation compared to those without Q80K (70.6–85.5% vs 55.0–66.7%) [38,59]. Currently, however, there is still limited information available regarding the impact of Q80K on Gt1b HCV infection, despite the fact that the effect of Q80K has been well characterized for Gt1a. In the first place, the epidemiology and characteristics of Gt1 sequences having the Q80K/R variant should be further studied, as searches of the Los Alamos database yielded a very unsatisfying number of previously identified sequences (S3 and S4 Tables). Detailed examination of the linkage between genotype and several RAVs may provide additional insights the clinical relevance of low-frequency genotype, drug-resistant quasispecies and their impact on the DAA therapy outcome.

Similarly to all studies, this study has some limitations. Firstly, the number of cases studied was very small, thus, the statistical power was insufficient to certainly detect low-prevalence mutations (e.g., R155, A156, and D168) if present. Secondly, although randomly selected, there might be a certain bias in enrolling HCV mono-infecting samples with a history of blood transfusion available. In this study, sample information including age, sex, associated illness, and source of infection, was not taken into consideration in the analysis; thus, the possibility of confounding and selection biases still remains. Thirdly, this study does not include hemophiliacs with HCV infection without HIV coinfection, because of sample unavailability. Finally, since this is a single-time point observational study, no information on the dynamic evolution of viral quasispecies is available. We are currently in process of another study targeting NS3 and NS5A using paired serum samples of pre-therapy, post-therapy, and post-relapse for hemophiliacs previously treated with peg-IFN plus ribavirin. We will also conduct post-trial surveillance of DAAs including simeprevir and sofosbuvir, wherein the NS3 and NS5B would be the target regions.

In conclusion, we developed and validated novel genotyping and RAV screening pipelines for HCV using the emerging technologies of NGS and QSR, reinforcing their potentials for the deconvolution of low-frequency genotypes, RAVs, and their interrelationships. Our study clearly demonstrated how the compositions of pre-existing minor genotypes and RAVs are considerably different between hemophiliacs and nonhemophiliacs, and HCV monoinfected patients with or without a history of whole-blood transfusion. These results strongly warrant further studies investigating the epidemiology and impacts of low-frequency variants on the clinical outcome of DAA therapies among hemophiliacs and other high-risk populations.

## Supporting Information

S1 Fig Pairwise SNV-to-SNV distance distributions of HCV core and NS3 protease region estimated from Los Alamos HCV reference sequences. (A, B) SNV-to-SNV distance distributions of all possible Gt pairs of (A) core and (B) NS3 protease region were estimated from aligned reference sequences obtained from Los Alamos HCV sequence database. (C-F) Intragenotype (C, D) and intrasubtype (E, F) SNV-to-SNV distance distributions of (C, E) core and (D, F) NS3, respectively. A white notch represents median, and a red bar represents mean in each box-whisker chart.
(TIF)

S2 Fig Phylogenetic positions of reconstructed sequences assigned to false-positive genotypes. Normalized patristic distances from reference sequences of each Gt were averaged, and distances from Gt1b and Gt2a were plotted. Sequences assigned to Gt1b were depicted in blue; Gt2a in cyan; Gt1a in red; Gt2b in orange; Gt2k in purple.
(TIF)

S3 Fig Quantitative accuracy of different QSR methods for detecting minor genotypes. Reconstructed abundances under different simulation conditions were paired with corresponding preset abundance values. The conditions tested were as follows: (A, C) QuRe-Low and QuRe-High, and (B, D) QuasiRecomb-Low and QuasiRecomb-High, wherein Low represents the total read count of 30,000, and High represents 100,000 for (A, B) core and (C, D) NS3 protease region (NS3). Note that the abundance threshold was set to 0.001, and values below 0.001 were replaced with 0.001 for descriptive purposes.
(TIF)

S4 Fig NS3 PI RAVs reproducibly detected by QuRe. QSR was performed using QuRe, and relative abundances of resistance-associated variants (RAVs) in the NS3 protease region were estimated in each subject. The x-axis labels are sample IDs colored on the basis of their history of exposure to blood (see Table 1 for details). The y-axis RAV labels were colored on the basis of the effects of RAVs on simeprevir susceptibility: susceptible (FC < 2) substitutions are in cyan, moderately resistant substitutions (2 < FC < 50) in magenta. No highly resistant substitutions (FC > 50) were detected. The threshold was set at a frequency of 0.0001.
(TIF)

S5 Fig NS3 PI RAVs reproducibly detected by QuasiRecomb. QSR was performed using QuasiRecomb, and relative abundances of resistance-associated variants (RAV) in the NS3 protease region were estimated in each subject. The x-axis labels are sample IDs colored on the basis of their status of exposure to blood (see Table 1 for details). The y-axis RAV labels are colored on the basis of the effects of RAVs on simeprevir susceptibility: susceptible (FC < 2) substitutions are in cyan, moderately resistant substitutions (2 < FC < 50) in magenta; highly resistant substitutions (FC > 50) are in brown. The threshold was set at a frequency of 0.0001.
(TIF)

S1 Table Primers used for RT-PCR. (XLSX)

S2 Table Parameter settings for QSR simulations. (XLSX)

S3 Table Counts of NS3 amino acid at position 80 binned by genotype in reported sequences retrieved from the Los Alamos HCV sequence database. (XLSX)

S4 Table Counts of NS3 amino acid at position 80 binned by sampled country in reported sequences retrieved from the Los Alamos HCV sequence database. (XLSX)
