# Supplementary figures and images for: Deconvoluting the Composition of Low-Frequency Hepatitis C Viral Quasispecies: Comparison of Genotypes and NS3 Resistance-Associated Variants between HCV/HIV Coinfected Hemophiliacs and HCV Monoinfected Patients in Japan
Source: PLoS One. 2015 Mar 6;10(3):e0119145. doi: 10.1371/journal.pone.0119145 (PMC4351984; doi:10.1371/journal.pone.0119145)

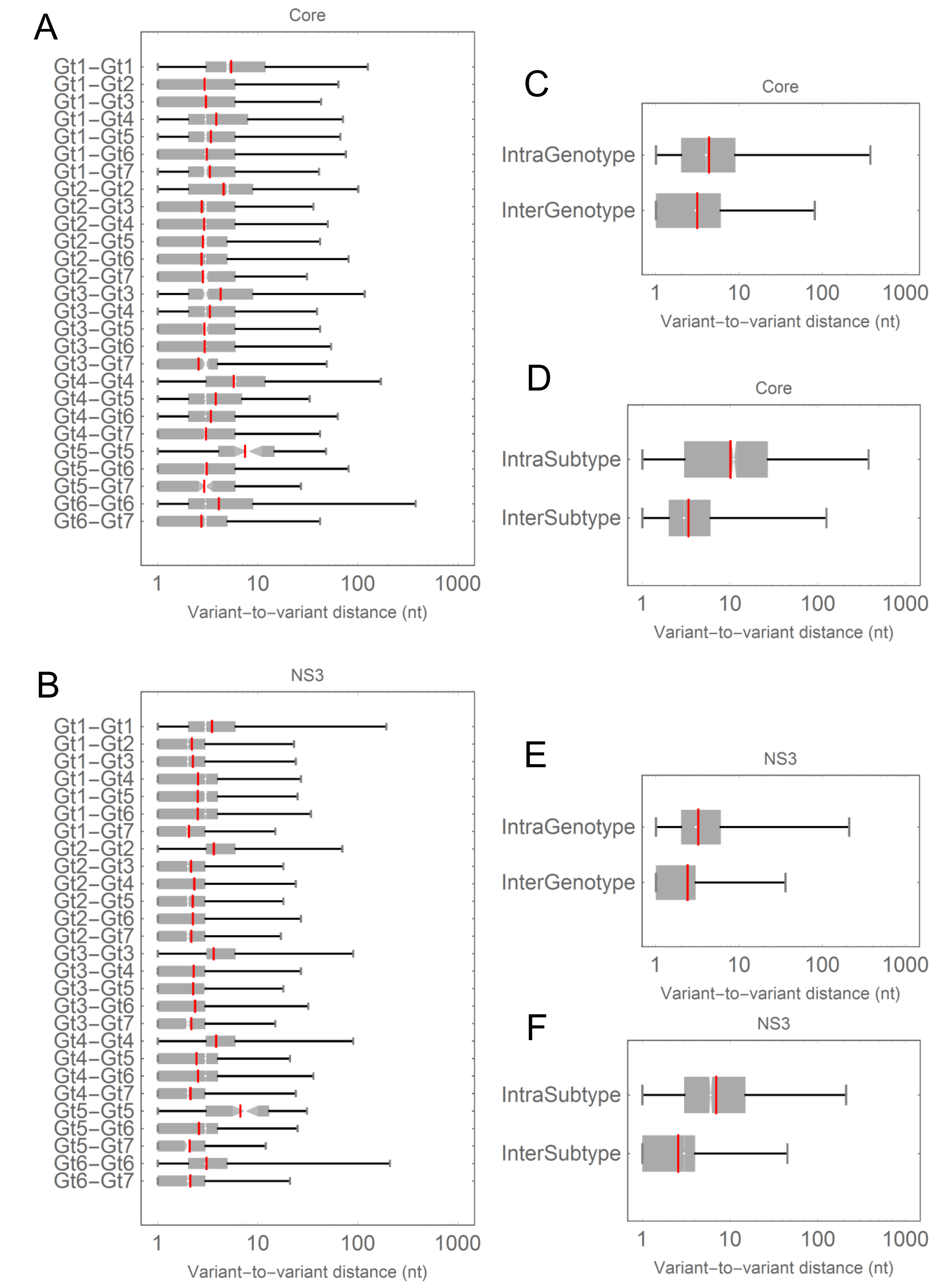

Supplement: S1 Fig — (A, B) SNV-to-SNV distance distributions of all possible Gt pairs of (A) core and (B) NS3 protease region were estimated from aligned reference sequences obtained from Los Alamos HCV sequence database. (C-F) Intragenotype (C, D) and intrasubtype (E, F) SNV-to-SNV distance distributions of (C, E) core and (D, F) NS3, respectively. A white notch represents median, and a red bar represents mean in each box-whisker chart. (TIF) [file pone.0119145.s001.tif]

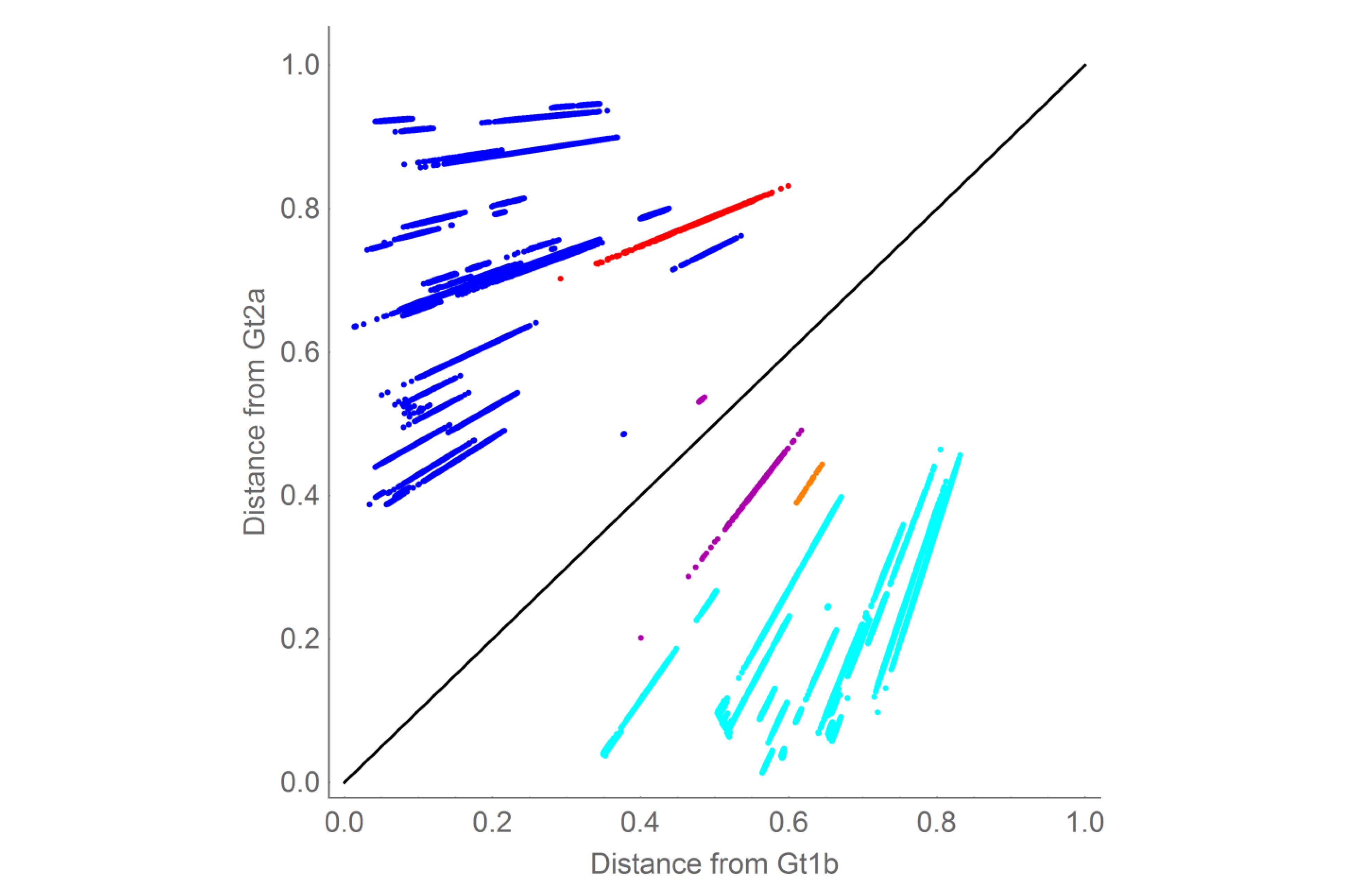

Supplement: S2 Fig — Normalized patristic distances from reference sequences of each Gt were averaged, and distances from Gt1b and Gt2a were plotted. Sequences assigned to Gt1b were depicted in blue; Gt2a in cyan; Gt1a in red; Gt2b in orange; Gt2k in purple. (TIF) [file pone.0119145.s002.tif]

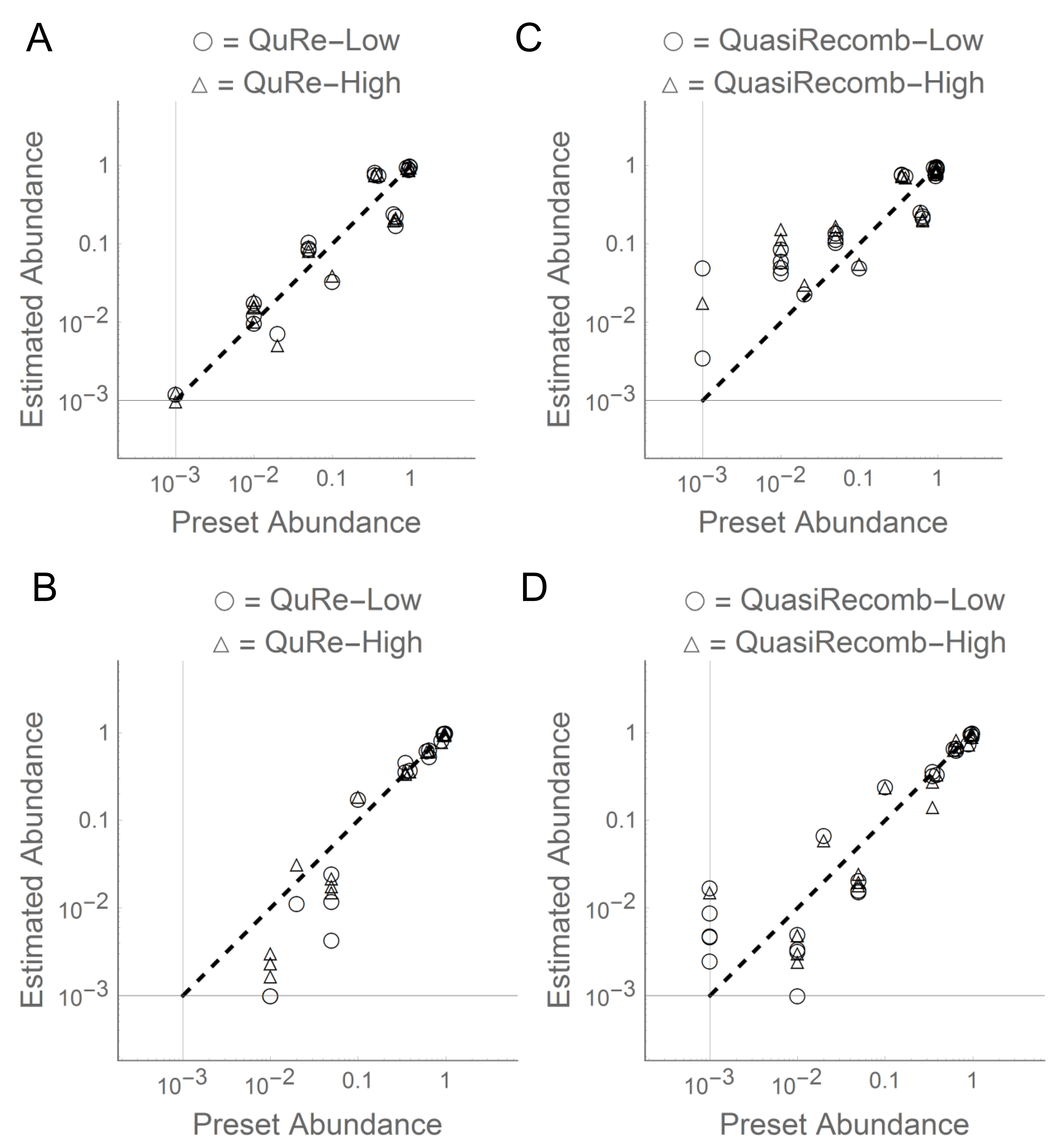

Supplement: S3 Fig — Reconstructed abundances under different simulation conditions were paired with corresponding preset abundance values. The conditions tested were as follows: (A, C) QuRe-Low and QuRe-High, and (B, D) QuasiRecomb-Low and QuasiRecomb-High, wherein Low represents the total read count of 30,000, and High represents 100,000 for (A, B) core and (C, D) NS3 protease region (NS3). Note that the abundance threshold was set to 0.001, and values below 0.001 were replaced with 0.001 for descriptive purposes. (TIF) [file pone.0119145.s003.tif]

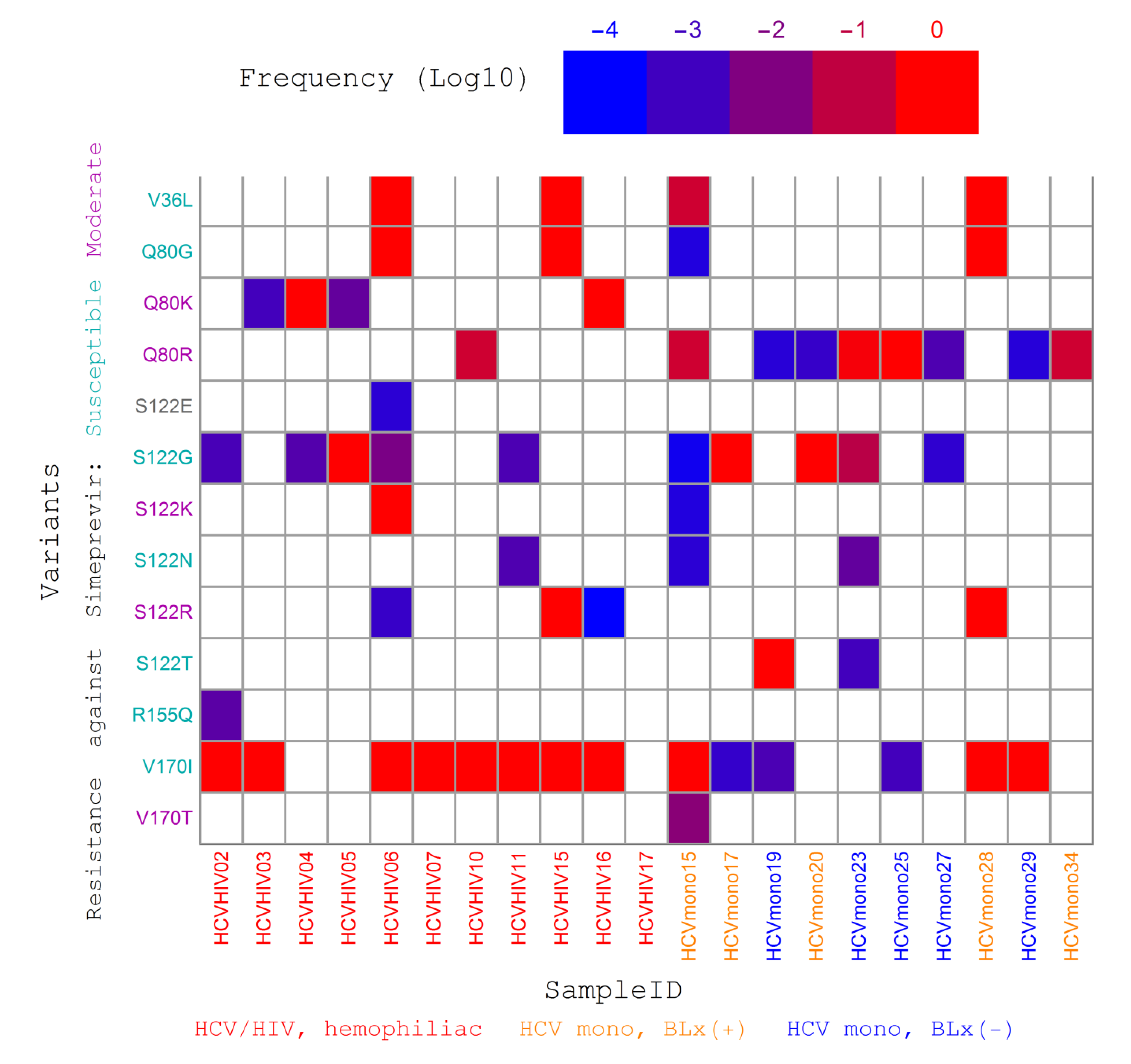

Supplement: S4 Fig — QSR was performed using QuRe, and relative abundances of resistance-associated variants (RAVs) in the NS3 protease region were estimated in each subject. The x-axis labels are sample IDs colored on the basis of their history of exposure to blood (see Table 1 for details). The y-axis RAV labels were colored on the basis of the effects of RAVs on simeprevir susceptibility: susceptible (FC < 2) substitutions are in cyan, moderately resistant substitutions (2 < FC < 50) in magenta. No highly resistant substitutions (FC > 50) were detected. The threshold was set at a frequency of 0.0001. (TIF) [file pone.0119145.s004.tif]

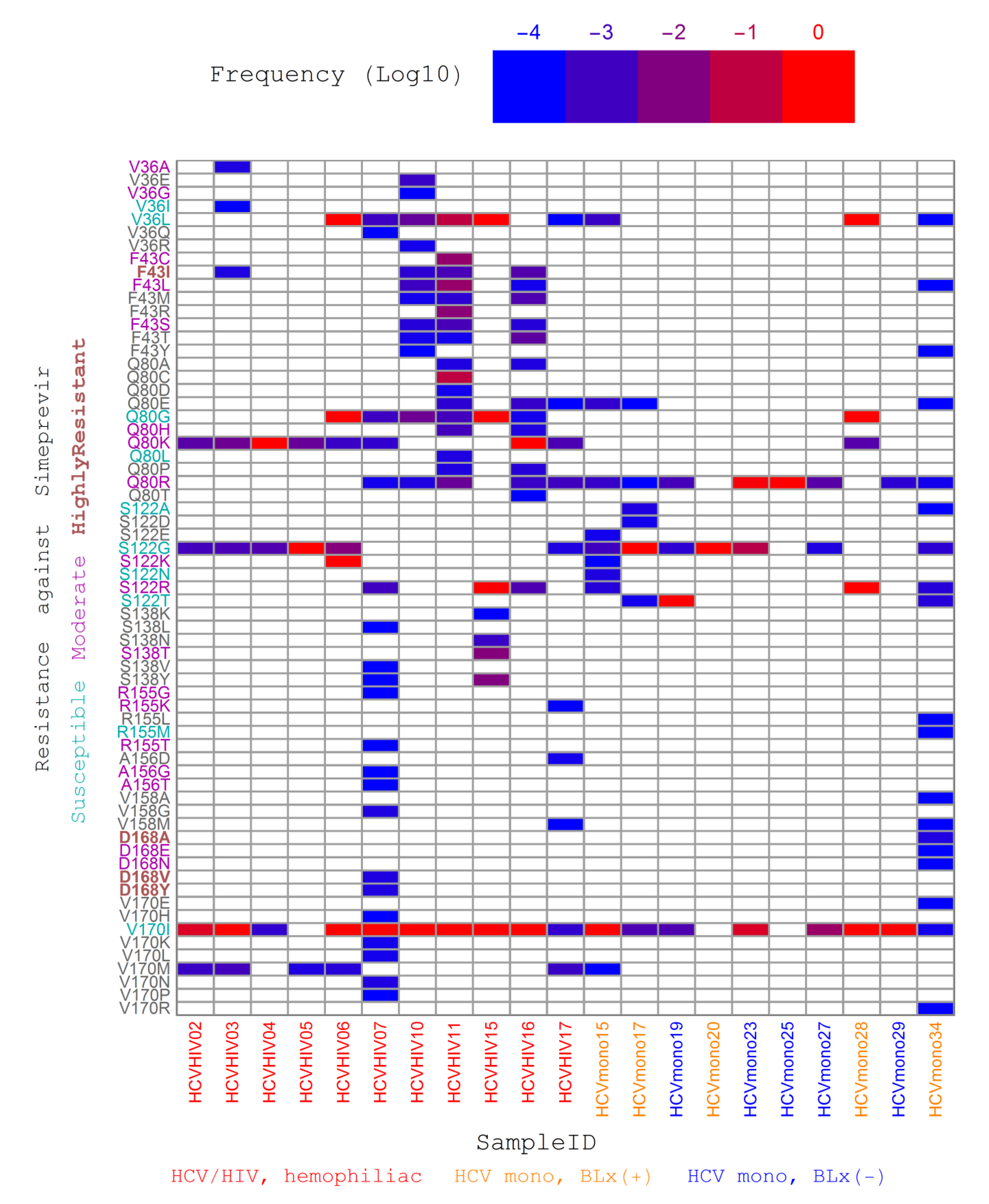

Supplement: S5 Fig — QSR was performed using QuasiRecomb, and relative abundances of resistance-associated variants (RAV) in the NS3 protease region were estimated in each subject. The x-axis labels are sample IDs colored on the basis of their status of exposure to blood (see Table 1 for details). The y-axis RAV labels are colored on the basis of the effects of RAVs on simeprevir susceptibility: susceptible (FC < 2) substitutions are in cyan, moderately resistant substitutions (2 < FC < 50) in magenta; highly resistant substitutions (FC > 50) are in brown. The threshold was set at a frequency of 0.0001. (TIF) [file pone.0119145.s005.tif]
